# Chamber studies of OH + dimethyl sulfoxide and dimethyl disulfide: insights into the dimethyl sulfide oxidation mechanism

**DOI:** 10.5194/acp-24-1299-2024

**Published:** 2024-01-30

**Authors:** Matthew B. Goss, Jesse H. Kroll

**Affiliations:** 1 Department of Civil and Environmental Engineering, Massachusetts Institute of Technology, Cambridge, Massachusetts 02139, USA; 2 Department of Chemical Engineering, Massachusetts Institute of Technology, Cambridge, Massachusetts 02139, USA

## Abstract

The oxidation of dimethyl sulfide (DMS) in the marine atmosphere represents an important natural source of non-sea-salt sulfate aerosol, but the chemical mechanisms underlying this process remain uncertain. While recent studies have focused on the role of the peroxy radical isomerization channel in DMS oxidation, this work revisits the impact of the other channels (OH addition and OH abstraction followed by bimolecular RO_2_ reaction) on aerosol formation from DMS. Due to the presence of common intermediate species, the oxidation of dimethyl sulfoxide (DMSO) and dimethyl disulfide (DMDS) can shed light on these two DMS reaction channels; they are also both atmospherically relevant species in their own right. This work examines the OH oxidation of DMSO and DMDS, using chamber experiments monitored by chemical ionization mass spectrometry and aerosol mass spectrometry to study the full range of sulfur-containing products across a range of NO concentrations. The oxidation of both compounds is found to lead to rapid aerosol formation (which does not involve the intermediate formation of SO_2_), with a substantial fraction (14%–47 % S yield for DMSO and 5 %–21 % for DMDS) of reacted sulfur ending up in the particle phase and the highest yields observed under elevated NO conditions. Aerosol is observed to consist mainly of sulfate, methanesulfonic acid, and methanesulfinic acid. In the gas phase, the NO_*x*_ dependence of several products, including SO_2_ and S_2_-containing organosulfur species, suggest reaction pathways not included in current mechanisms. Based on the commonalities with the DMS oxidation mechanism, DMSO and DMDS results are used to reconstruct DMS aerosol yields; these reconstructions roughly match DMS aerosol yield measurements from the literature but differ in composition, underscoring remaining uncertainties in sulfur chemistry. This work indicates that both the abstraction and addition channels contribute to rapid aerosol formation from DMS and highlights the need for more study into the fate of small sulfur radical intermediates (e.g., CH_3_S, CH_3_SO_2_, and CH_3_SO_3_) that are thought to play central roles in the DMS oxidation mechanism.

## 1 Introduction

Dimethyl sulfide (DMS; CH_3_SCH_3_) represents an important biogenic contribution to atmospheric sulfur. Through its oxidation in the troposphere, it acts as the dominant source of non-sea-salt sulfate aerosol over the oceans and, as such, may affect the climate system through direct (aerosol–radiation) and indirect (aerosol–cloud) effects. Thus, understanding DMS-derived aerosol formation and properties is important for understanding the natural background climate state (Carslaw et al., 2013; Fung et al., 2022), as well as forecasting climate changes in the future. The detailed chemistry of DMS oxidation determines the yield of aerosol and the ultimate fate of the sulfur, but despite decades of research (Yin et al., 1990a; Barnes et al., 2006; Hoffmann et al., 2016) and notable recent breakthroughs (Wu et al., 2015; Berndt et al., 2019; Veres et al., 2020), the underlying chemical mechanism is not fully understood.

The impacts of DMS-derived aerosol are affected by several chemical factors, including the total aerosol yield, the timescale of aerosol formation, and the aerosol composition. All of these factors may affect the net aerosol radiative impact (Fung et al., 2022), and all are directly controlled by secondary chemistry, much of which remains uncertain. Sulfate from gas-phase DMS oxidation is thought to form through several channels. One involves the formation and subsequent oxidation of SO_2_, which is relatively slow: SO_2_ + OH life-time ≈ 12 d (assuming [OH] = 10^6^ molec.cm^−3^, 1 atm, and 298 K; Burkholder et al., 2020); for context, SO_2_ lifetime to all atmospheric losses ≈ 1.4 d (Fung et al., 2022). Alternatively, some channels may lead to the direct formation of SO_3_, which rapidly converts to sulfuric acid in the presence of water vapor, providing a potentially faster path to sulfate aerosol. This direct-formation route has been known for decades (Bandy et al., 1992; Lucas and Prinn, 2002), is regularly included in chemical mechanisms describing DMS oxidation (Saunders et al., 2003; Barnes et al., 2006; Wollesen de Jonge et al., 2021; Fung et al., 2022), and has been demonstrated in a number of laboratory studies (Shen et al., 2022; Ye et al., 2022; Berndt et al., 2023). We refer to this pathway as “rapid aerosol formation”, defined as aerosol formation that does not involve SO_2_ as an intermediate species. The variability in timescale for aerosol formation may affect the spatial distribution and amount of secondary sulfate aerosol in the atmosphere and may, as a result, affect radiative impacts (Fung et al., 2022). Sulfate can also be produced in the aqueous phase, and so the balance between gas- and aqueous-phase sulfate-formation pathways may impact total new particle formation (Hodshire et al., 2019). Mechanisms also control aerosol composition, additionally influencing aerosol properties and impact. Aerosol-phase products of DMS consist mostly of sulfate/sulfuric acid and methanesulfonic acid (MSA) (Barnes et al., 2006), and while both can contribute to new particle formation (Hodshire et al., 2019), these species are likely to nucleate at different rates (Chen et al., 2016; Hodshire et al., 2019).

**Figure 1.**
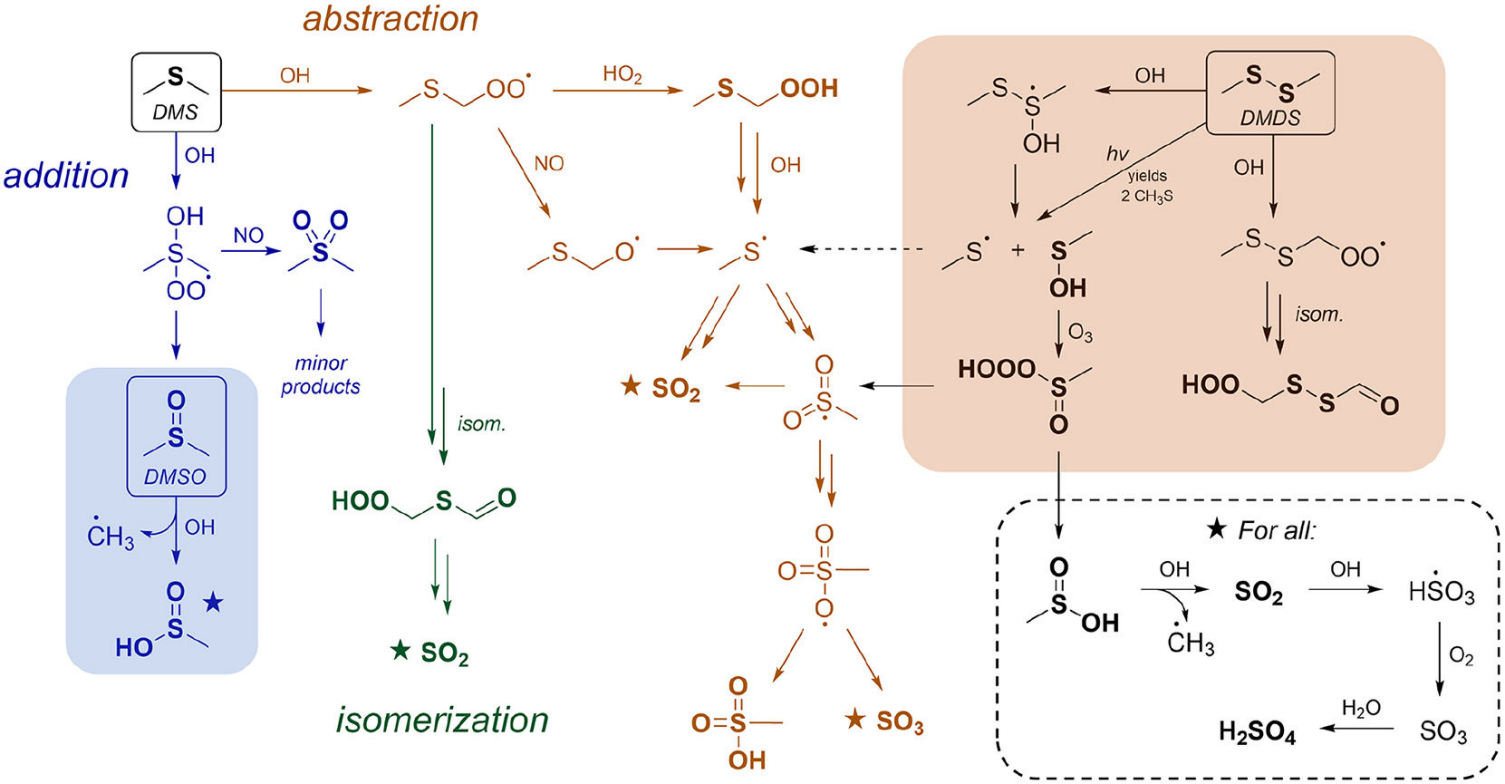
Simplified gas-phase oxidation scheme for DMS, DMSO, and DMDS. From the top left: DMS oxidation (Barnes et al., 2006; Wu et al., 2015; Veres et al., 2020), in which three major channels (addition, abstraction, and isomerization shown in blue, orange, and green, respectively) control product distributions. The shaded blue box shows the oxidation of DMSO (Burkholder et al., 2020), which represents an important intermediate in the DMS OH addition channel. The shaded orange box shows the oxidation of DMDS (Berndt et al., 2020), which overlaps with DMS oxidation through the formation of CH_3_S, a key radical intermediate in the DMS OH abstraction channel. Further reaction of species marked with a star is shown in the dashed box. Compounds in bold represent closed-shell species. Under this scheme, rapid aerosol formation (which does not involve the intermediate formation of SO_2_) occurs only via the abstraction channel. More complete schemes are given in Barnes et al. (2006), Hoffmann et al. (2016), Ye et al. (2022), and Berndt et al. (2023), as well as in Figs. 4 and S15.

The oxidation of DMS by OH is characterized by three main pathways: OH addition, OH abstraction followed by bimolecular reaction of the RO_2_ radical, and OH abstraction followed by RO_2_ isomerization (referred to from here on as addition, abstraction, and isomerization, respectively). These are shown in Fig. 1, which features a simplified oxidation mechanism for DMS. Recent work has focused largely on the isomerization channel (Wu et al., 2015; Berndt et al., 2019; Veres et al., 2020; Ye et al., 2021, 2022; Novak et al., 2021; Jernigan et al., 2022; Assaf et al., 2023), since it represents a major revision of the traditional oxidation mechanism, accounting for 30 %–46 % of the total DMS fate globally (Veres et al., 2020; Novak et al., 2021; Fung et al., 2022). However, the major product of the isomerization channel, hydroperoxymethyl thioformate (HPMTF), is thought not to contribute to rapid aerosol formation and is instead thought to oxidize mainly to SO_2_ or be lost to clouds (Vermeuel et al., 2020; Novak et al., 2021).

In this study, we focus on the other two channels (abstraction and addition) for which significant uncertainties remain, particularly with respect to their relative contributions to rapid aerosol formation. Under the scheme from the Master Chemical Mechanism (MCM 3.3.1) (Saunders et al., 2003) and the Jet Propulsion Laboratory (JPL) kinetics recommendations (Burkholder et al., 2020), the abstraction channel is almost solely responsible for rapid aerosol formation (Fig. 1). In our recent work, we showed that a modified version of the MCM scheme accurately predicts total aerosol yields as measured in chamber experiments but dramatically underpredicts measured MSA (Ye et al., 2022). Other studies have also noted discrepancies in MSA production between measurements and model predictions (Lucas and Prinn, 2002; von Glasow and Crutzen, 2004; Wollesen de Jonge et al., 2021; Shen et al., 2022). This has led to some suggested changes in the mechanism, most notably a modification to the oxidation of methanesulfinic acid (MSIA), leading to the formation of a radical intermediate (MSIA + OH → CH_3_SO_2_ + H_2_O), which can then react further to generate MSA (Lucas and Prinn, 2002; von Glasow and Crutzen, 2004; Barnes et al., 2006; Wollesen de Jonge et al., 2021; Ye et al., 2022; Shen et al., 2022). This change allows for rapid aerosol formation from the addition channel and improves the model-mechanism agreement substantially in some cases (Wollesen de Jonge et al., 2021; Shen et al., 2022) but not others (Ye et al., 2022). Despite these developments, the relative importance of the abstraction and addition channels for aerosol formation remains poorly constrained.

Here, we investigate the above uncertainties via the oxidation of two related compounds, dimethyl sulfoxide (DMSO; CH_3_S(O)CH_3_) and dimethyl disulfide (DMDS; CH_3_SSCH_3_). These each have reaction channels in common with the addition and abstraction branches of the DMS mechanism (shaded areas in Fig. 1). DMSO is a key intermediate in the DMS addition channel, and so its oxidation (shown in blue in Fig. 1) provides insight into that channel’s product formation and aerosol formation. Similarly, DMDS oxidation (shown in orange in Fig. 1) forms the CH_3_S radical as a major intermediate. This radical is thought to be a key intermediate in the DMS abstraction channel, leading to the formation of SO_2_, MSA, and sulfate. These two precursors therefore allow relatively independent access to two of the major branches of the DMS oxidation mechanism, allowing us to investigate product formation, including rapid aerosol production, from each branch. Beyond their direct relevance to DMS, both species are important in their own right. DMDS is emitted directly from marine (Kilgour et al., 2022), biomass burning (Berndt et al., 2020), and agricultural sources (Filipy et al., 2006; Trabue et al., 2008; Rumsey et al., 2014) and is estimated to represent a few percent of biogenic sulfur emissions (Tyndall and Ravishankara, 1991), while DMSO has been observed in measurable concentrations in the marine boundary layer (Berresheim et al., 1993; Bandy et al., 1996; Nowak et al., 2001).

Past experimental study of DMSO oxidation has shown significant variability in product distributions, with relatively little study of aerosol formation. Most prior studies were carried out before the widespread adoption of the aerosol mass spectrometer (AMS) or chemical ionization mass spectrometer (CIMS) and generally apply spectroscopic methods (Barnes et al., 1989; Sørensen et al., 1996; Urbanski et al., 1998; Arsene et al., 2002; Librando et al., 2004) or offline ion chromatography (IC) (Sørensen et al., 1996; Arsene et al., 2002; Librando et al., 2004; Chen and Jang, 2012). While studies generally agree that MSIA is the dominant first-generation oxidation product (Arsene et al., 2002; Barnes et al., 2006), the yields of other products have been inconsistent, with SO_2_ reported as a major (Sørensen et al., 1996; Kukui et al., 2003; Librando et al., 2004; Chen and Jang, 2012) or a minor (Arsene et al., 2002) product and highly variable yields of MSA (< 0.5 %–34 %) (Sørensen et al., 1996; Arsene et al., 2002; Librando et al., 2004; Chen and Jang, 2012) and dimethyl sulfone (DMSO_2_, 2.9 %–33 %) (Sørensen et al., 1996; Arsene et al., 2002; Librando et al., 2004; Chen and Jang, 2012). The wide variability in the reported product yields may be due to several factors: high starting concentrations (> 1 ppm, parts per million) (Barnes et al., 1989; Sørensen et al., 1996; Arsene et al., 2002; Librando et al., 2004) may favor RO_2_-RO_2_ reactions; setups that do not allow for aerosol measurements (Barnes et al., 1989; Urbanski et al., 1998; Kukui et al., 2003) may underestimate the yields of more oxidized products; and experiments carried out in nitrogen atmospheres (Kukui et al., 2003) may not promote RO_2_ chemistry. While offline IC methods (Arsene et al., 2002; Librando et al., 2004; Chen and Jang, 2012) detected aerosol products, to our knowledge only two previous studies (Chen and Jang, 2012; Van Rooy et al., 2021a) have examined aerosol production from DMSO using realtime techniques.

Similar to DMSO, relatively few recent studies have examined the products from DMDS oxidation, and only one study has characterized aerosol-phase products using online measurements. Early work (Yin et al., 1990b; Barnes et al., 1994) reports SO_2_ as the major product (~ 80 %~90 % yield under low NO_*x*_ and lower at high NO_*x*_); MSA and H_2_SO_4_ are reported as minor products (0 %–11 %, increasing with increasing NO_*x*_) (Yin et al., 1990b). These findings are in agreement with newer studies that find that aerosol concentrations increase with increasing NO_*x*_ and that the ratio of MSA to H_2_SO_4_ depends on the oxidant and relative humidity (Van Rooy et al., 2021a, b). Recently, CIMS studies by Berndt et al. (2020, 2023) found low yields of MSA and MSIA, evidence of gas-phase formation of H_2_SO_4_, and evidence of a minor (~ 2 %) OH abstraction channel, leading to the formation of HOOCH_2_SSCHO via isomerization (right side of Fig. 1). While prior studies have established a mechanism that largely explains laboratory observations of gas-phase products (Berndt et al., 2020), the mechanism of aerosol formation has yet to be thoroughly explored.

In this work, we conduct chamber experiments to study the OH oxidation of DMSO and DMDS under different NO_*x*_ conditions (lower NO and higher NO), measuring the products with an AMS and CIMS. This study seeks not only to assess the relative aerosol yield and composition from DMSO and DMDS oxidation but also to evaluate these results in the context of DMS oxidation to better understand the role of the abstraction and addition channels in rapid aerosol formation.

## 2 Methods

All experiments were run in a 7.5 m^3^ environmental chamber (Hunter et al., 2014) operated in “semi-batch” mode, in which clean air was added to replace air sampled by the instruments (chamber dilution lifetime ≈ 8.9 h). Ultraviolet lights centered at ~ 340 nm illuminated the chamber (*J*_NO_2__ ≈ 0.06 min^−1^); only 50 % of lights were used for the OH oxidation of DMDS to slow down oxidation chemistry. All experiments were run at 20 °C and < 5 % relative humidity, providing conditions that should prevent multiphase chemistry. This allows this work to focus on gas-phase oxidation processes and facilitates comparison with prior studies, most of which were also carried out under dry, room-temperature conditions.

For each experiment, dry sodium nitrate seed particles were atomized into the chamber using an aerosol generator (TSI model 3076) and a diffusion dryer (Brechtel), providing condensation nuclei that can be easily distinguished from secondary sulfate. For DMDS experiments, the seed solution was washed with dichloromethane to remove any organic compounds from the solution. To additionally probe the influence of dichloromethane for DMSO oxidation, 600 ppb (parts per billion) dichloromethane was added to a single experiment (expt 1) at *t* = 1.92 h and was not observed to affect product formation. For lower-NO experiments (defined as experiments with no added source of NO_*x*_; estimated background NO ≈ 10 ppt (parts per trillion) in the presence of H_2_O_2_ and UV light (Ye et al., 2022)), the OH precursor hydrogen peroxide (H_2_O_2_) was added via a direct injection of a known volume of 30 % H_2_O_2_ solution into the main chamber dilution airflow. Lower-NO experiments were run first in each series of experiments to reduce the influence of possible residual NO_*x*_. For higher-NO experiments (defined as experiments with an added source of NO_*x*_; total [NO_*x*_] > 20 ppb, with [NO] varying over the course of the experiment; see the Supplement), the OH precursor nitrous acid (HONO) was generated by mixing 10 mL 0.06 M sodium nitrite with 10 mL 0.05 M sulfuric acid and introduced to the chamber by flowing a stream of clean air through the headspace for 20–50 s. Additional NO is introduced to the chamber as a byproduct of this reaction. The flask containing the sulfuric acid and NaNO_2_ solution was left connected to the chamber after the airflow was stopped, allowing for slow continued diffusion of HONO into the chamber; the degree of diffusion varied between experiments (see the Supplement for NO_*x*_ data). Previous chamber experiments suggest that reaction with O(^3^P) can contribute to the oxidation of reduced sulfur compounds (Van Rooy et al., 2021a); however, this is likely to be negligible under the lower-NO_2_ and lower-UV conditions used here (see the Supplement). DMDS and DMSO (Sigma-Aldrich; > 99.0 %) were introduced through the heated inlet (80 and 150 °C, respectively) via syringe injection. For some experiments (1 and 5), NO_*x*_ conditions were perturbed by the addition of NO or HONO after several hours of oxidation. Acetonitrile (0.07 μL; 4.5 ppb) was added to the chamber for use as a dilution tracer, since its loss due to reaction with OH is negligible on the timescale of these experiments. Conditions for each experiment are shown in Table 1.

Concentrations of precursors and products were monitored via a suite of online instrumentation. DMDS was monitored using a gas chromatograph with flame ionization detection (GC-FID; SRI Instruments). DMSO, acetonitrile, and oxidized gas-phase products were measured using an ammonium chemical ionization mass spectrometer (${\text{NH}}_{4}^{+}$ CIMS; modified PTR3; see Zaytsev et al., 2019). For DMSO experiments, the initial DMSO addition was found to overwhelm the primary ion in the ${\text{NH}}_{4}^{+}$ CIMS. This was avoided by diluting the flow into the CIMS by a factor of ~ 14. This dilution factor was quantified by adding the acetonitrile tracer to the chamber before the dilution flow was started and measuring the change in the acetonitrile signal. Particlephase products were quantified using an aerosol mass spectrometer (Aerodyne Research Inc., HR-ToF-AMS; abbreviated as AMS from here on) and scanning mobility particle sizer (SMPS; TSI models 3080 and 3775). Additional gas monitors measured sulfur dioxide (Teledyne T100), ozone (2BTech Model 202), and NO/NO_2_ (Thermo Scientific Model 42i). Initial HONO concentration was estimated based on the NO_2_ channel in the NO_*x*_ monitor; since NO_2_ may have also been present, this represents an upper limit.

The concentrations of gas-phase species were calculated based on direct calibration where possible and voltage scanning where reference standards were not available. For DMSO, the ${\text{NH}}_{4}^{+}$ CIMS sensitivity was directly calibrated using a liquid calibration unit (Ionicon Analytik). One experiment (expt 4) was carried out 2 weeks before the calibration, and the sensitivity was re-scaled based on the change in the primary ion concentration. While most oxidized products showed smooth time series, the DMSO signal (C_2_H_6_SO(${\text{NH}}_{4}^{+}$)) was somewhat unstable, suggesting inconsistent detection, which may introduce additional uncertainty into this measurement. The sensitivity of the GC-FID to DMDS was calculated based on known volumes added to the chamber. For all other gas-phase organics detected by the ${\text{NH}}_{4}^{+}$ CIMS, concentrations were derived using voltage scanning, following the methods described in Zaytsev et al. (2019). Gas-phase quantification methods are described in further detail in the Supplement.

Quantification of particle-phase products using the AMS followed a new method developed to distinguish different S-containing aerosol components (sulfate, methanesulfonate, and methanesulfinate). In brief, reference AMS spectra were taken for ammonium methanesulfonate and sodium methanesulfinate atomized directly into the AMS. Organosulfur peaks from the experimental AMS data are fit as a linear combination of the same organosulfur peaks from the two reference spectra. These two factors explain the experimental organosulfur peaks well (median *r*^2^ ≈ 0.95; Fig. S2). Based on this, MSIA and MSA factors are subtracted out, leaving a residual sulfate signal and a small organic residual. These factors are converted to mass using the relative ionization efficiencies (RIEs) of the respective species. RIE values are directly calculated for sulfate and MSA (2.06; from the ammonium balance method; Hodshire et al., 2019); MSIA is assumed to have the same RIE as MSA, since it cannot be directly calculated via the same method without the ammonium MSIA salt. As discussed below, there is some ambiguity in the particle-phase MSIA assignment, especially for the DMDS experiments; given this uncertainty, we denote this species MSIA*. This assignment, and the AMS quantification methods generally, are described in greater detail in the Supplement.

All gas-phase species were corrected for dilution loss by dividing by a normalized exponential fit of the acetonitrile time series. Aerosol-phase products are corrected for dilution, wall loss, and any changes in the collection efficiency over time by normalizing to the high-resolution nitrate time series from the seed particles (Eq. 4 from Wang et al., 2018). The wall- and dilution-corrected AMS signal is then scaled, such that the initial seed aerosol concentration matches that measured by the SMPS.

## 3 Results and discussion

### 3.1 DMSO oxidation experiments

**Figure 2.**
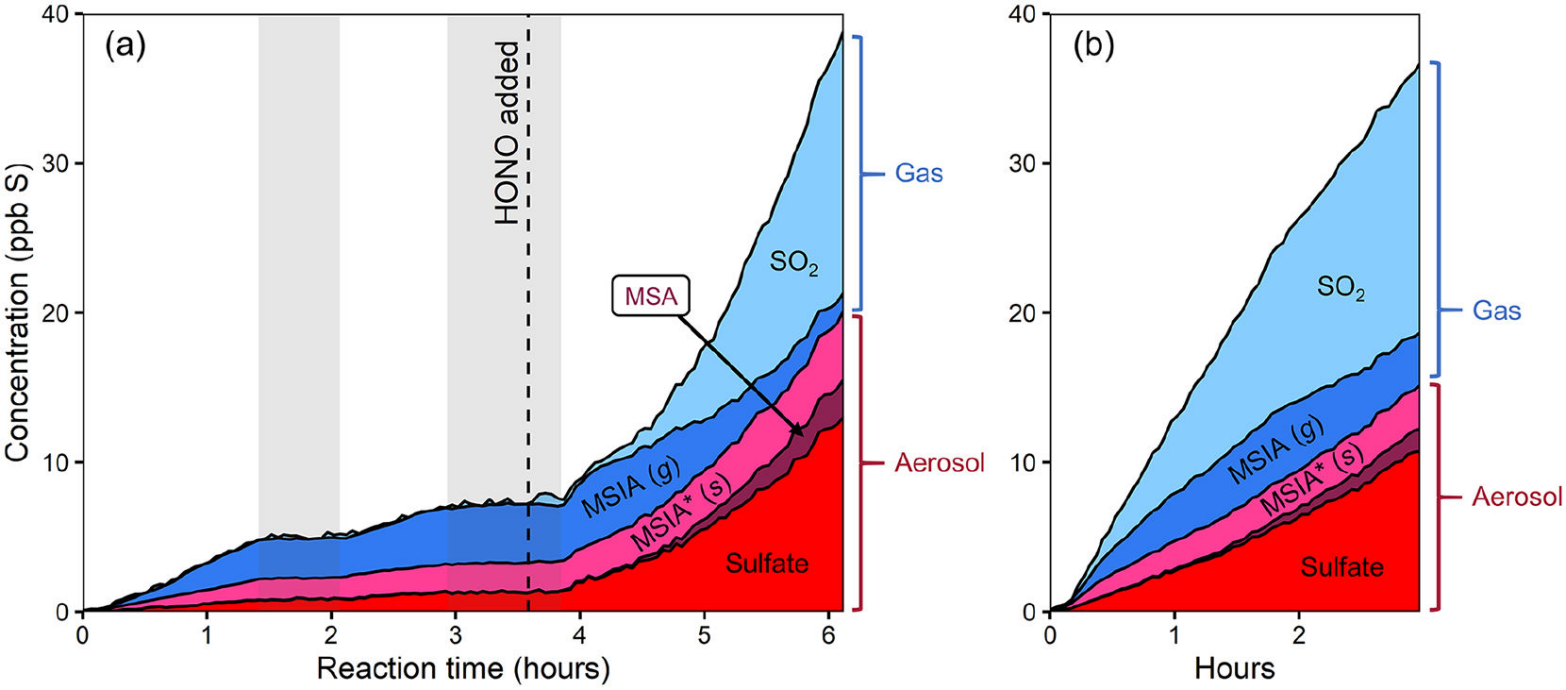
Stacked product time series from the oxidation of DMSO, using different oxidant precursors. **(a)** Experiment 1, with H_2_O_2_ followed by HONO addition after several hours. **(b)** Experiment 2, with HONO. Production of particle-phase products increases dramatically in the presence of NO_*x*_. The light gray bars in panel **(a)** indicate when the chamber lights were turned off for diagnostic purposes. The lower-NO period is dominated by MSIA production, while the higher-NO conditions show large increases in the concentrations of SO_2_, MSA, and sulfate. The product distribution is comparable in both higher-NO conditions.

Figure 2 shows stacked time series of oxidation products for two DMSO experiments. In experiment 1 (Fig. 2a), DMSO is initially oxidized with H_2_O_2_ as the oxidant precursor and no added NO_*x*_. Halfway through the experiment, HONO is added, substantially increasing both total NO_*x*_ and OH concentrations (see Fig. S4 for NO_*x*_ time series). In experiment 2 (Fig. 2b), DMSO is oxidized with only HONO as an oxidant precursor. Due to some uncertainty in the DMSO time series, these plots focus only on the product composition; plots that include the DMSO time series are included in the Supplement. While sulfur closure appears complete in some experiments (Fig. S6), total sulfur drops over time during experiments using H_2_O_2_ as an oxidant precursor and briefly dips during HONO experiments. Incomplete sulfur closure may be due to a number of factors including the presence of unmeasured products, the loss of species via wall loss or other loss processes, error in CIMS sensitivity values (especially for DMSO), error in absolute particle-phase measurements, or error in the speciation of AMS data; as such, our discussion focuses primarily on trends in product formation and composition.

Under lower-NO conditions (first 3.5 h of expt 1; Fig. 2a), MSIA is the dominant product in the gas phase, and MSIA* the dominant product in the particle phase, with sulfate formed in low but nonzero yield. Notably, no SO_2_ or MSA are formed under these conditions (replicated in expt 3; Fig. S8a). Under higher-NO conditions, either from adding HONO to the ongoing experiment (last 2.5 h of expt 1; Fig. 2a) or from using HONO as the sole oxidant precursor (expt 2; Fig. 2b), the product distribution is dramatically different, with substantial production of MSA and sulfate in the particle phase and SO_2_ in the gas phase. All higher-NO experiments (expts 1, 2, and 4) exhibit consistent product distributions (see also Fig. S8b).

**Figure 3.**
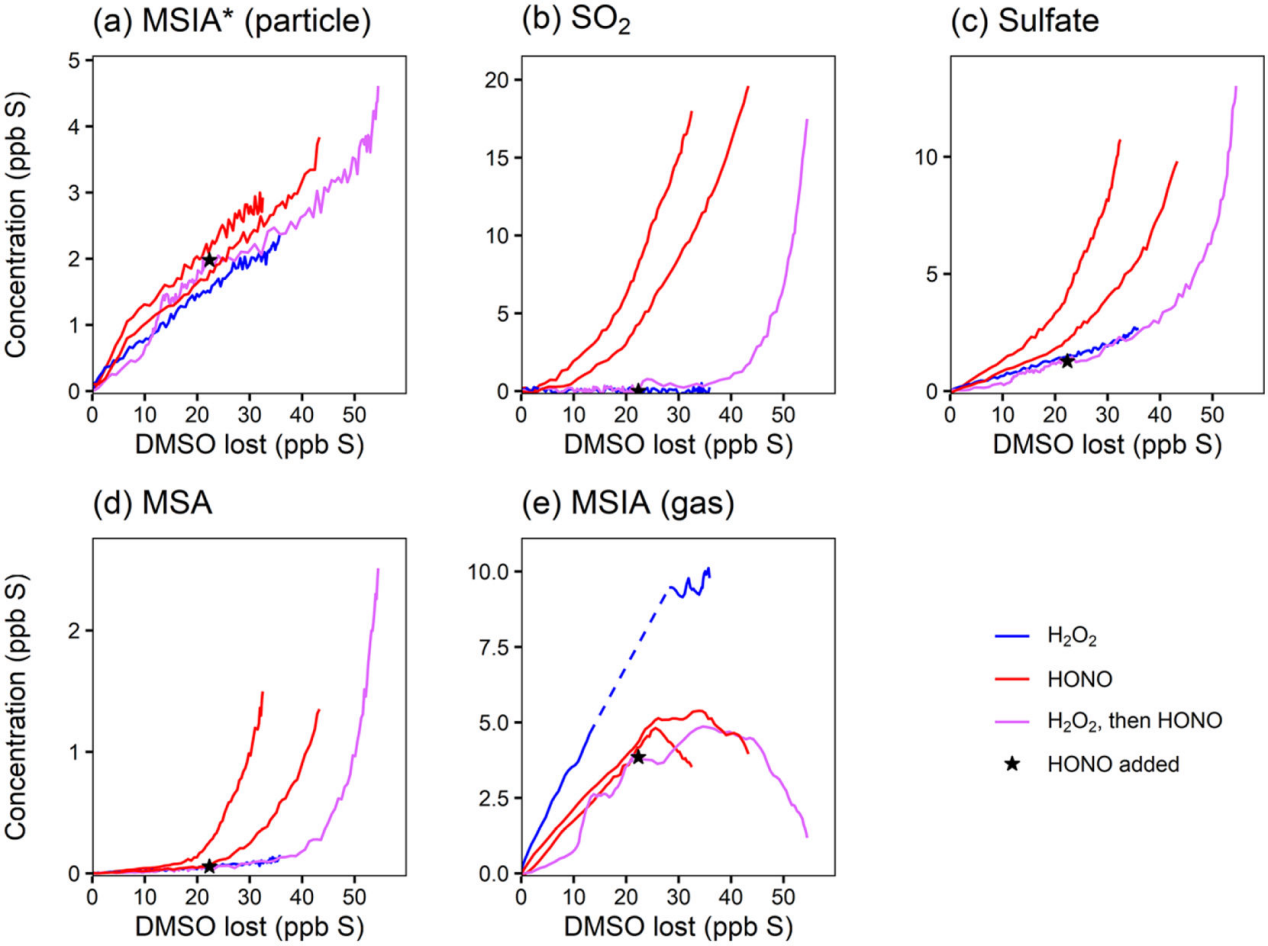
Yield plots for DMSO oxidation products. Major products are plotted against the loss of DMSO to normalize for changing OH concentrations and allow for comparisons among experiments 1–4. Colors refer to the oxidant precursor. For experiment 1 (pink), the NO_*x*_ regime is switched by adding HONO, as marked by the star. The dashed blue line indicates missing data. Note the differing *y* axes. Where traces lie on top of each other (e.g., for MSIA*; panel **a**), the addition of NO_*x*_ does not influence the chemistry. Where traces are distinct (e.g., for SO_2_; panel **b**), the product formation is influenced by NO_*x*_.

The use of HONO in experiments 1, 2, and 4 shifts the chemistry in two primary ways: the increase in NO changes the product branching ratios (i.e., by increasing RO_2_ + NO), and the increase in HONO and NO increases the OH concentration (directly through HONO photolysis and indirectly through HO_*x*_ cycling). To distinguish these two effects, the product time series are plotted against the amount of DMSO that has reacted away (Fig. 3), effectively normalizing for differing OH concentrations and allowing comparisons among experiments. To reduce the noise in these plots, the DMSO time series used as the basis for the *x* axes are smoothed using a penalized spline (see the Supplement). Any uncertainties in [DMSO] from unstable detection in the ${\text{NH}}_{4}^{+}$ CIMS and possible run-to-run variability in the calibration factor manifest as uncertainty in the x axis in these plots; this likely explains the majority of the x offset in the duplicate experiments (red traces) (see also Fig. S12). As such, these plots cannot distinguish small changes in product yields but should still show major differences in yields.

Figure 3a shows that the MSIA* yield is unchanged by the different experimental conditions, suggesting that its formation from DMSO + OH is independent of NO_*x*_. This is consistent with the literature mechanism, which involves OH addition followed by loss of the CH_3_ radical (Fig. 1). This mechanism suggests that MSIA should form in 100 % yield in the first generation of oxidation, which should involve an initial total MSIA slope of 1; the lower slope seen here (Fig. 3a and e) may be a result of incomplete sulfur closure (Fig. S6) and possible uncertainty in the speciation ascribed to AMS data. In contrast to MSIA*, SO_2_ (Fig. 3b) shows a large shift in yield at a given OH exposure for higher vs. lower NO_*x*_, suggesting that NO_*x*_ plays a role in its formation; this is inconsistent with the literature mechanisms (Fig. 1). Sulfate and MSA (Fig. 3c and d) are intermediate cases; barring significant error in the DMSO calibration (factor of ~ 1.5–2), they appear moderately dependent on NO_*x*_ concentrations. Gas-phase MSIA concentrations start to decrease (Fig. 3e), even as particle-phase MSIA* concentrations continue to grow (Fig. 3a); this suggests that MSIA may experience slower oxidation in the particle phase under these conditions, such that aerosol particles serve as a reservoir for this species.

In addition to using HONO to perturb the system, one lower-NO experiment (expt 3) is perturbed by the addition of O_3_ to investigate the impact of the CH_3_SO_2_ + O_3_ → CH_3_SO_3_ + O_2_ reaction (Barnes et al., 2006) (Fig. S8a). Since CH_3_SO_3_ is thought to be a major intermediate leading to the formation of sulfate and MSA, the addition of ozone is expected to influence the formation of particle-phase products. That no change in product distribution is observed upon the addition of O_3_ suggests that the CH_3_SO_2_ + O_3_ reaction is slow or that CH_3_SO_2_ is not formed from the reaction under these conditions.

While the range of products detected (SO_2_, MSIA/MSIA*, MSA, and sulfate) is broadly consistent with those found in previous DMSO oxidation studies (Barnes et al., 1989; Sørensen et al., 1996; Urbanski et al., 1998; Arsene et al., 2002; Librando et al., 2004; Chen and Jang, 2012), differences in NO_*x*_ dependence and aerosol composition stand out. The strong increase in SO_2_ formation with increased NO_*x*_ has not been reported in previous studies, possibly due to the range of NO_*x*_ concentrations used. While some studies (Barnes et al., 1989; Sørensen et al., 1996) were run with ppm levels of NO_*x*_, exceptions include Librando et al. (2004), whose low-NO_*x*_ case was < 20 ppb, which may not be sufficiently low to see evidence of this chemistry, and Arsene et al. (2002), who used synthetic air to obtain low-NO conditions and saw a minor shift in the SO_2_ yield. Previous studies on the dependence of MSA formation on NO_*x*_ levels are inconsistent, with some (Sørensen et al., 1996; Arsene et al., 2002) showing no dependence and others (Chen and Jang, 2012) showing an increase in MSA with higher initial NO concentrations. The results from Chen and Jang (2012) are in better agreement with our measurements, though their reported MSA / sulfate ratio is substantially different (this work reports 0.14 : 1 to 0.19 : 1 at elevated NO_*x*_; Chen and Jang, 2012, find ~ 2.7 : 1 to ~ 10 : 1 at elevated NO_*x*_), possibly influenced by their higher-NO concentrations and higher-RH conditions (fostering aqueous chemistry). While MSIA has been measured as a major first-generation product, it has not previously been measured in the particle phase, though the exact speciation of aerosol-phase compounds detected by the AMS carries some uncertainty (see the Supplement). Sulfate, with yields ranging from ~ 6 % in lower-NO conditions to ~ 27 % in higher-NO conditions, has been quantified in only one other study (Chen and Jang, 2012), where it is seen in lower yield (~ 2 %–4 %). Under the conditions in our chamber (dry; [OH] = 3.7 × 10^5^ to 2.7 × 10^6^ molec. cm^−3^), the SO_2_ lifetime to OH oxidation is > 100 h, and heterogeneous oxidation of SO_2_ is unlikely, implying that the observed sulfate is not formed from SO_2_. This indicates that our observed formation of sulfate formation is via a rapid aerosol-formation mechanism, likely involving the direct formation of SO_3_.

In contrast to some previous studies (Sørensen et al., 1996; Arsene et al., 2002; Librando et al., 2004; Chen and Jang, 2012), we did not observe DMSO_2_ as a product. A small DMSO_2_ signal appeared when DMSO was added to the chamber, but it did not grow with oxidation and so was likely an impurity in the DMSO or an artifact from the CIMS detection of DMSO. Most previous studies that detected DMSO_2_ as a product were run at ppm levels of DMSO (Sørensen et al., 1996; Arsene et al., 2002; Librando et al., 2004) and so may have been influenced by bimolecular reactions such as DMSO + RO_2_ reactions (Arsene et al., 2002), which are less likely to occur under lower-concentration conditions. Similar to DMSO_2_, methanesulfonyl peroxynitrate (MSPN; CH_3_S(O)_2_OONO_2_), which has previously been detected (Sørensen et al., 1996; Arsene et al., 2002; Librando et al., 2004), was not observed. This might be because MSPN is not detectable with ${\text{NH}}_{4}^{+}$ CIMS or because of the lower-NO_*x*_ levels used; in our experiments, total NO_*x*_ was ~ 50 ppb, far lower than the > 1 ppm levels used in some previous studies (Arsene et al., 2002; Librando et al., 2004). No other products were observed in the ${\text{NH}}_{4}^{+}$ CIMS. This supports prior assertions that OH abstraction from the methyl groups of DMSO is too slow to compete (González-García et al., 2006), since we observed no products that would be expected from the resulting peroxy radicals (e.g., from RO_2_ + HO_2_).

**Figure 4.**
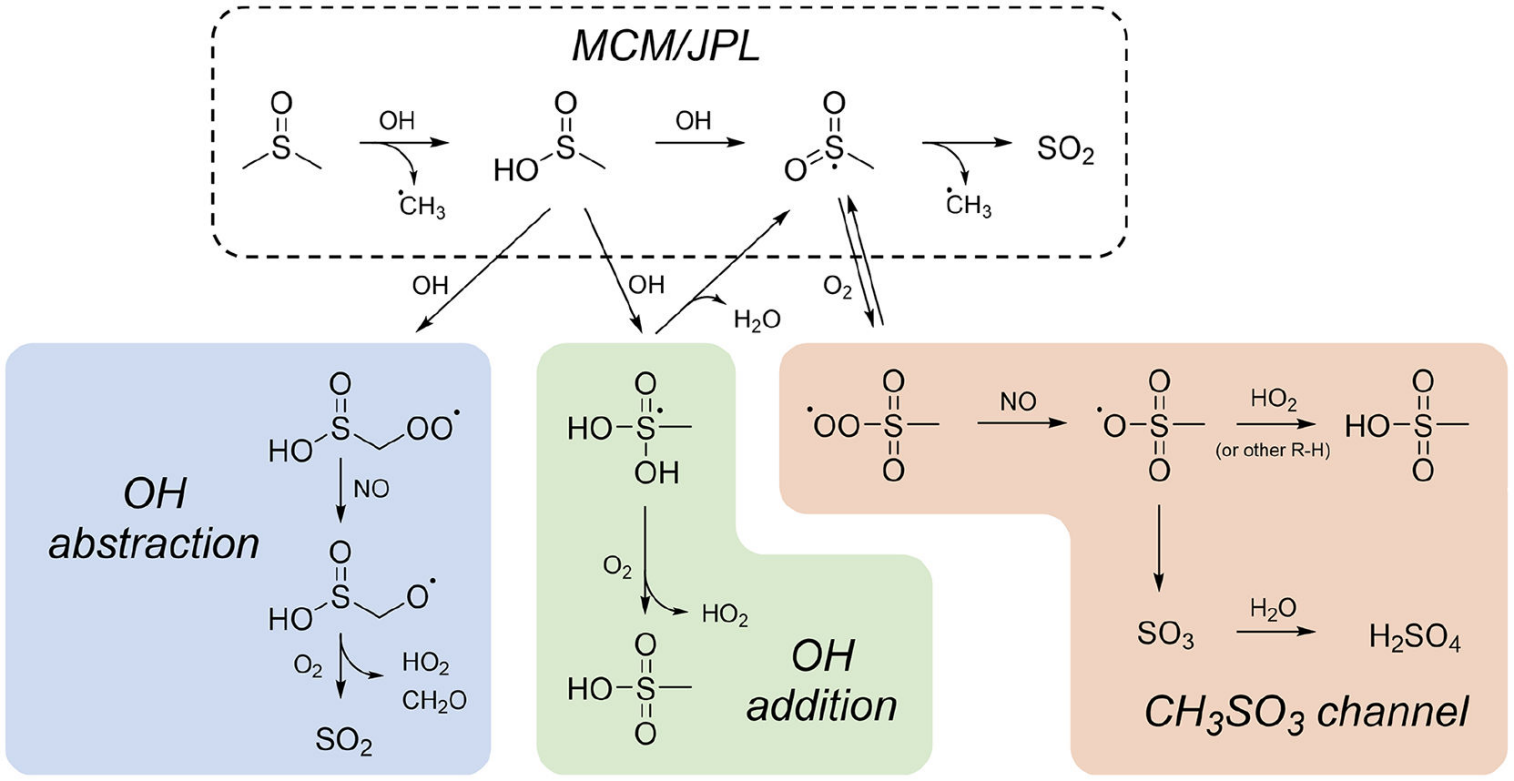
Proposed mechanisms for DMSO and MSIA oxidation. The mechanism recommended by JPL and used in the MCM (dashed box) involves the formation of SO_2_ only. The OH abstraction pathway (blue) proceeds via OH abstraction of a methyl hydrogen from MSIA, leading to the formation of SO_2_. The OH addition pathway (green) proceeds via OH addition to the S atom of MSIA, leading to the formation of MSA. The CH_3_SO_3_ channel (orange) proceeds via O_2_ addition to the CH_3_SO_2_ radical and leads to the formation both MSA and sulfate via the CH_3_SO_3_ radical. Estimated rates for these reactions and box model simulation results are included in the Supplement.

The observations above suggest a need to revise the standard DMSO oxidation mechanism, as recommended by JPL (Burkholder et al., 2020) and included in the MCM (Saunders et al., 2003). Figure 4 shows this mechanism (dashed box) in addition to other possible mechanisms. In the JPL/MCM mechanism, DMSO reacts with OH to form MSIA, which reacts with OH to form SO_2_ in unit yield. However, this is inconsistent with our observation of rapid sulfate and MSA formation and the lack of SO_2_ formation at lower NO_*x*_. The shaded boxes in Fig. 4 show three possible alternative pathways, all of which involve modification to the MSIA oxidation mechanism. Pathways that do not involve MSIA formation have been shown to be unlikely (González-García et al., 2006); this is consistent with our lack of detection of products such as DMSO_2_ or CH_3_S(O)CH_2_OOH. Estimated rates for reaction pathways shown in Fig. 4, as well as box model simulations that demonstrate the effects of these pathways using the Framework for 0-D Atmospheric Modeling (F0AM; Wolfe et al., 2016), can be found in the Supplement (Table S2 and Fig. S16).

In the CH_3_SO_3_ channel, the CH_3_SO_2_ intermediate (formed from abstraction of the acidic hydrogen of MSIA) does not fall apart to CH_3_ and SO_2_, as in the JPL/MCM mechanism, but rather reacts with O_2_ to form more oxidized products (Lucas and Prinn, 2002). This channel has recently received renewed attention (Wollesen de Jonge et al., 2021; Shen et al., 2022; Ye et al., 2022), since it provides a pathway to both MSA and sulfate. However, under higher-NO conditions, where measured MSA and sulfate yields are highest, the HO_2_ concentration is suppressed. Since HO_2_ + CH_3_SO_3_ is the final reaction leading to MSA, this mechanism can sometimes underpredict MSA (Ye et al., 2022). However, recent experimental evidence (Berndt et al., 2023) supports earlier hypotheses (Yin et al., 1990a; Barnes et al., 2006) that other hydrocarbons may serve as an H atom source for the CH_3_SO_3_ → CH_3_SO_3_H reaction. This could explain high MSA yields from chamber experiments, where the hydrocarbon concentration is typically much higher than in the atmosphere.

The other pathways shown, OH abstraction and OH addition, stem from possible products of the OH + MSIA reaction. The OH abstraction pathway represents a plausible explanation for the observation of SO_2_ formation at higher NO; however, OH abstraction of the methyl hydrogens is believed to be too slow to compete (Yin et al., 1990a; González-García et al., 2007). The OH addition channel represents a straightforward pathway to MSA (Shen et al., 2022) but is inconsistent with our observation that MSA forms in greatest yield at elevated [NO_*x*_]. While computational studies support this OH addition step as a minor pathway (Tian et al., 2007; González-García et al., 2007), they have not investigated the possibility of reaction with O_2_ to form MSA.

Several additional pathways to MSA (not shown) have been hypothesized but seem unlikely to be the major sources of MSA in our chamber experiments. Production of MSA via CH_3_SO_2_ + OH (Kukui et al., 2003; González-García et al., 2007) does not explain the observed NO_*x*_ dependence and seems unlikely due to low concentrations of both species. In addition, the disproportionation reaction of CH_3_SO_2_OO + RO_2_ may lead to MSA (Berndt et al., 2023), but this pathway is significant only when RO_2_ concentrations are sufficiently high to outcompete other pathways. In our chamber, this reaction can occur under lower-NO conditions, where a small amount of MSA is formed, but it is likely only a minor contributor to MSA production under higher-NO conditions, when the majority of MSA is formed (see modeling results in the Supplement). Based on observations of NO_*x*_ and humidity dependence, Van Rooy et al. (2021b) suggest that CH_3_SO_3_ may react with NO or NO_2_ to form CH_3_S(O)_2_ONO or CH_3_S(O)_2_ONO_2_ before reacting with water to form MSA and HNO_3_ or HONO. We did not observe the nitrite or nitrate compound, and the subsequent hydrolysis step is unlikely under the dry conditions in our chamber.

The observed trends in product formation, particularly the formation of MSA and sulfate and the lack of SO_2_ formation under lower-NO_*x*_ conditions, make clear that the commonly used JPL/MCM mechanism of DMSO oxidation is inadequate; however, none of the above-proposed mechanisms discussed above is fully consistent with computational and laboratory results. In box model simulations of these pathways (see Sect. S9), we are unable to reproduce all of the experimental results presented here (especially the NO_*x*_ dependence of SO_2_ formation). More computational and experimental studies on the fate of MSIA and radical intermediates (e.g., CH_3_SO_2_ and CH_3_SO_3_) are thus necessary to better constrain this oxidation mechanism.

### 3.2 DMDS oxidation experiments

**Figure 5.**
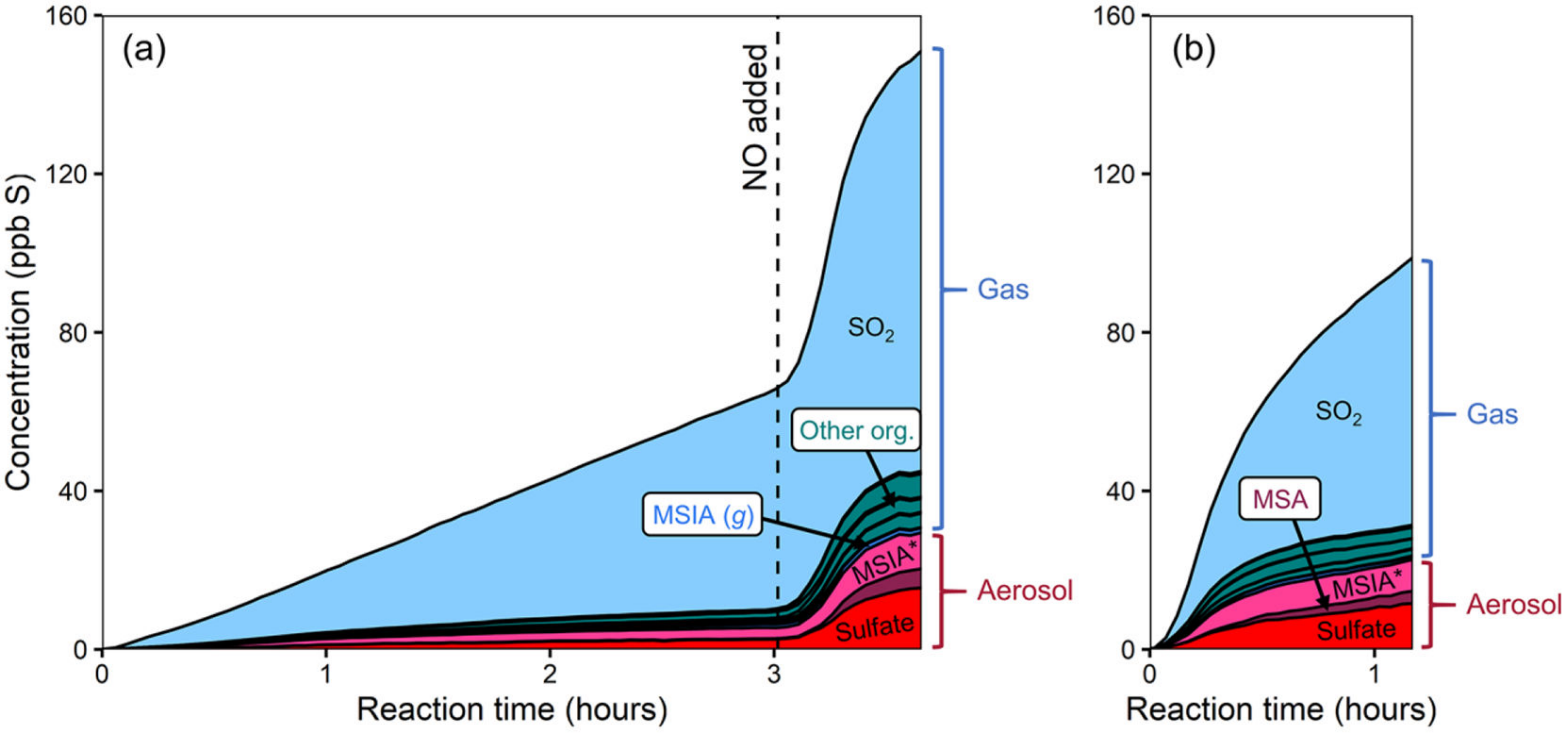
Stacked product time series from the oxidation of DMDS, using different oxidant precursors. **(a)** Experiment 5, with H_2_O_2_ followed by NO addition after several hours. **(b)** Experiment 6, with HONO. All gas-phase organic compounds detected by the ${\text{NH}}_{4}^{+}$ CIMS, other than MSIA (*g*), are shown in green and shown in greater detail in Fig. 7. SO_2_ is the major product formed in both experiments, but other species increase under higher NO. Product distributions are similar under both higher-NO cases (right side of panel **a**; **b**).

Figure 5 shows stacked time series for the products of two DMDS oxidation experiments. In Fig. 5a (experiment 5), DMDS is oxidized using H_2_O_2_ as the OH precursor (lower-NO conditions); after 3 h, NO is added, increasing total NO_*x*_ and OH concentrations. Figure 5b shows the products of experiment 6, where DMDS was oxidized using HONO as an oxidant precursor. Plots that include the DMDS time series are included in the Supplement.

In both higher- and lower-NO conditions, oxidation products (Fig. 5) are dominated by SO_2_, though a range of other gas- and particle-phase products are also formed. As in the DMSO experiments, aerosol formation increases substantially in the presence of NO_*x*_, and MSA is formed only after the addition of NO_*x*_. Increased NO_*x*_ also increases the production of organic products detected by the ${\text{NH}}_{4}^{+}$ CIMS. The product distributions of the two higher-NO cases (expts 5 and 6) are consistent. Direct photolysis of DMDS also occurs to some extent during each experiment. To explore this, DMDS was exposed to twice the light intensity of the other DMDS experiments (expt 7; Fig. S9) and formed almost entirely SO_2_, suggesting that this may bias SO_2_ yields from OH oxidation of DMDS. Based on the SO_2_ yield from photolysis, photolytically derived SO_2_ is estimated to make up 6 %–20 % of the SO_2_ generated in the OH oxidation experiments.

One clear difference between the DMSO and DMDS product distributions is the apparent partitioning of MSIA/MSIA* between the gas and particle phase (for DMSO 36 ± 13 % (1σ) particle-phase; for DMDS 91 ± 8% (1σ) particle-phase; see Figs. 2 and 5). The reason for this difference is not clear. Different particle-phase acidity could affect partitioning, with lower pH driving more MSIA to the gas phase. The discrepancy may also be a result of ambiguity in the AMS spectra, where some organosulfur species, including those with two sulfur atoms, are likely to contribute to the same AMS peaks as MSIA.

**Figure 6.**
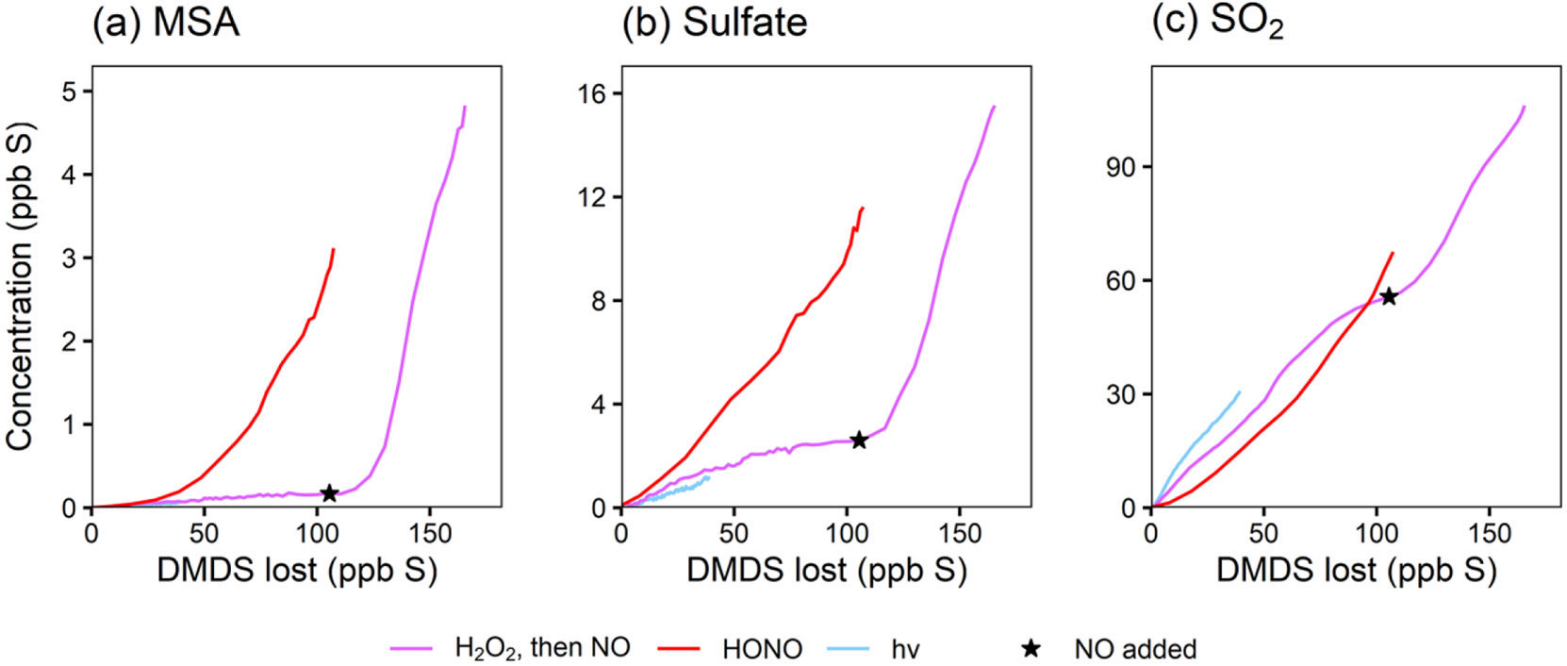
Yield plots for selected DMDS oxidation products. MSA, sulfate, and SO_2_ are plotted against the loss of DMDS to normalize for changing OH concentrations and therefore allow comparisons among experiments 5–7. Colors denote experimental conditions. For one experiment (expt 5; pink trace), the NO_*x*_ regime is switched by adding NO, as marked by the star. Note the differing *y* axes. Where traces lie on top of each other (e.g., for SO_2_), the addition of NO_*x*_ does not influence the chemistry. Where traces are distinct (e.g., for MSA and sulfate), the product formation is influenced by NO_*x*_. See Fig. S13 for similar plots of other products.

As done previously for DMSO, selected DMDS products for experiments 5–7 are plotted against DMDS loss to normalize for changing [OH] and allow for direct comparisons among experiments (Fig. 6). These plots demonstrate dramatic increases in yield for particle-phase species (MSA, sulfate, and MSIA*; see the Supplement) under high-NO_*x*_ conditions. This is consistent with recent measurements of increased production of gas-phase MSA and H_2_SO_4_ (Berndt et al., 2023) and increased production of particle-phase products when NO_*x*_ is added (Van Rooy et al., 2021a, b); though, in previous work (Van Rooy et al., 2021a), MSA formation was not observed under dry conditions. In contrast to the trends in particle-phase products, SO_2_ yields are relatively consistent among experiments, and exhibit no obvious dependence on NO_*x*_ concentrations, suggesting that the pathway leading to SO_2_ is different from that found in DMSO oxidation. These major products are largely consistent with literature mechanisms (Saunders et al., 2003; Barnes et al., 2006), where a high yield of CH_3_S provides multiple efficient routes to SO_2_ via O_2_ addition and rearrangement. The CH_3_SO_2_ radical, which can also form SO_2_, is in equilibrium with the CH_3_S(O)_2_OO radical, which can be diverted towards particle-phase products (MSA and sulfate) by reaction with NO, explaining the elevated aerosol yields at high NO_*x*_ (see Figs. 1 and 4). This might also explain the slightly lower SO_2_ yields in the HONO experiment. For the photolysis experiment (expt 7), the SO_2_ yield is slightly higher, likely due to the greater yield of CH_3_S radicals per molecule of DMDS.

**Figure 7.**
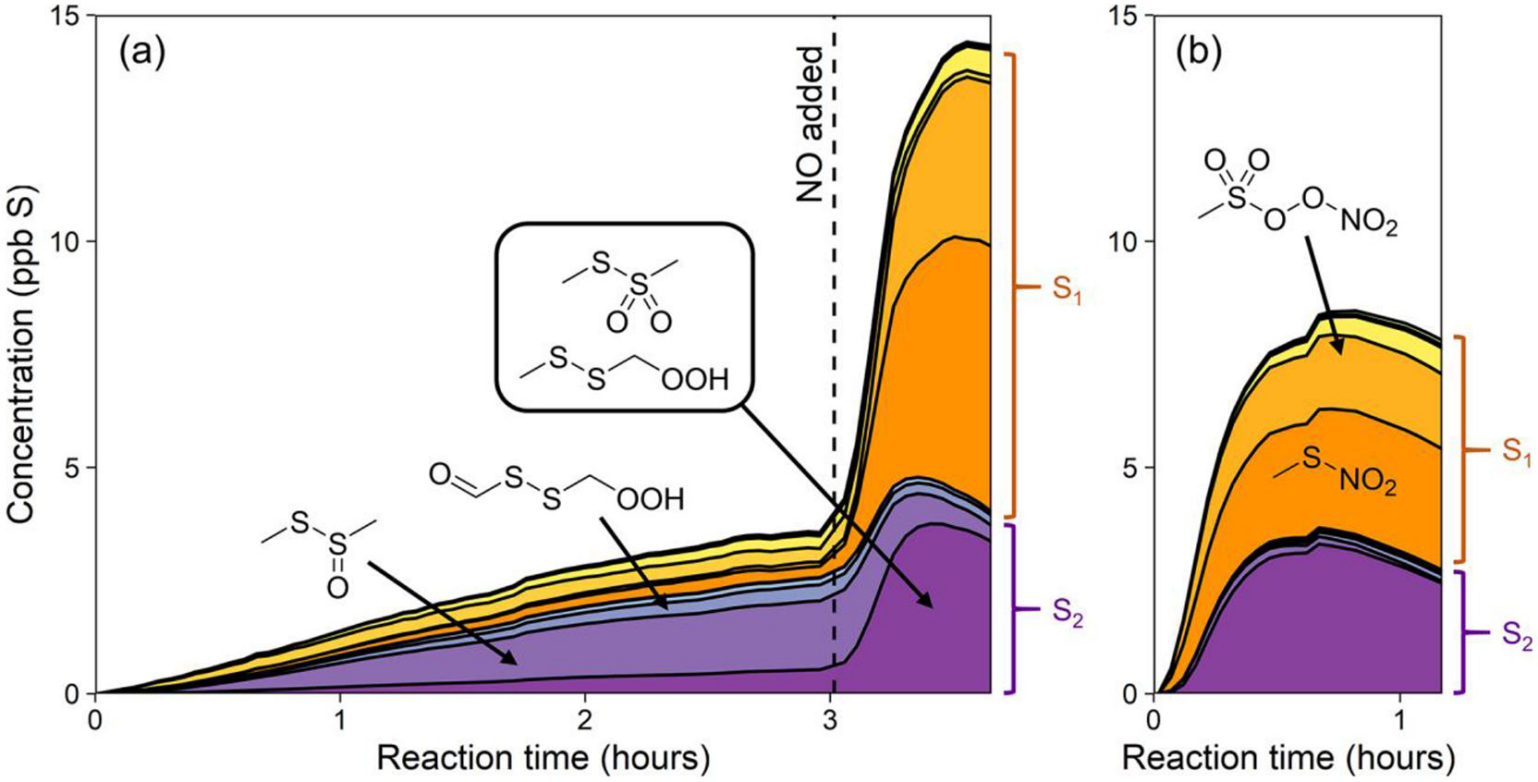
Stacked time series of minor gas-phase organosulfur products of DMDS oxidation for experiment 5 (**a**; H_2_O_2_ followed by NO) and experiment 6 (**b**; HONO). These are the products shown as “Other org.” in Fig. 5. Products are sorted into S_1_ (orange) and S_2_ (purple) compounds, and suggested structures of the most abundant products are shown. See Fig. S14 for full results.

Thus the major products of DMDS oxidation, including SO_2_, sulfate, and MSA, are explained reasonably well by known DMDS chemistry (Berndt et al., 2020) and CH_3_S chemistry, as understood from the DMS oxidation mechanism (Fig. 1). However, the detection of minor gas-phase organosulfur compounds, many containing two sulfur atoms, suggest additional minor reaction pathways. The time series of these “other organics” (shown in green in Fig. 5) are presented in Fig. 7. While S2 products are formed in low yield (~ 1 %–3 %), they may influence aerosol formation from DMDS due to their greater molecular weight and might contribute to the observed MSIA* product seen in the AMS.

Many of the observed organosulfur products are analogous to those formed in DMS oxidation, and include several previously unreported compounds, providing evidence of new DMDS reaction pathways. C_2_H_6_S_2_O is favored at lower NO and decays away after the addition of NO (Figs. 7a and S13b). Since the formation of an alcohol seems unlikely, this product is best explained by the structure CH_3_SS(O)CH_3_, a molecule analogous to DMSO and likely formed via the OH adduct (which is usually assumed to fragment into CH_3_S and CH_3_SO; Berndt et al., 2020). A complementary product, C_2_H_6_S_2_O_2_, forms mostly at higher NO (Figs. 7a-b and S13c). This is unlikely to be the hydroperoxide CH_3_SSCH_2_OOH, since that would likely be formed only at lower NO. Instead the product is better explained by the structure CH_3_SS(O)_2_CH_3_, which is similar to DMSO_2_ and likely also formed from the OH adduct. Together, these two compounds appear almost exactly analogous in structure and mechanism to the formation of DMSO and DMSO_2_ from the DMS–OH adduct and so represent a minor new oxidation pathway for DMDS. Also among the minor organosulfur products is C_2_H_4_S_2_O_3_, first detected by Berndt et al. (2020) and attributed to the isomerization product of the DMDS abstraction pathway (HOOCH_2_SSCHO; Figs. 1 and 7). This product is observed to form in greater yield at longer RO_2_ bimolecular lifetimes. At higher NO_*x*_, we observe CH_3_SO_6_N, likely methanesulfonyl peroxynitrate formed from CH_3_S(O)_2_OO and NO_2_, and CH_3_SNO_2_, likely formed from the reaction of CH_3_S and NO_2_. CH_4_SO_4_, postulated by Berndt et al. (2020) to be a source of MSIA, was not observed. The total mass spectrometric signal of gas-phase organics decreases slightly at the end of experiments, likely a result of further oxidation leading to fragmentation and/or condensation onto particles or chamber walls. A more detailed product time series figure (Fig. S14), hypothesized reaction mechanism (Fig. S15), and discussion of these species are given in the Supplement.

These chamber studies demonstrate several new observations of DMDS oxidation chemistry. The OH oxidation of DMDS leads to substantial rapid aerosol formation, with strong dependence on the NO_*x*_ regime (5 %–6 % S yield at lower NO_*x*_; 17 %–21 % S yield at higher NO_*x*_). In addition to the major products (SO_2_, sulfate, MSA, and MSIA), this work demonstrates that S_2_ species, formed through both OH abstraction and stabilization of the OH adduct, may represent a small but non-negligible fraction of the total product distribution, with a measured yield of ~ 3 % under higher-NO_*x*_ conditions.

### 3.3 Implications for DMS oxidation

As discussed in the introduction, the oxidation mechanisms of DMSO and DMDS overlap substantially with the DMS addition and abstraction channels, respectively, and can therefore be used to help interpret the contributions of these channels to aerosol formation from DMS. Our measurements show that DMSO and DMDS both produce aerosol in lower yield (final S yields of 14 %–15 % and 5 %–6 %, respectively) at lower NO and relatively high yield (final S yields of 34 %–47 % and 17 %–21 %) at higher NO, suggesting that both the addition and abstraction channels can be important contributors to rapid aerosol formation from DMS oxidation.

We can extrapolate the observations from DMSO and DMDS experiments based on the literature branching ratios to try to explain the rapid aerosol yields from DMS oxidation. Based on the JPL-recommended rates for abstraction and addition at 293 K (Burkholder et al., 2020), OH abstraction contributes 64 % of the DMS + OH reaction, while OH addition contributes the remaining 36 %. Within the addition channel, ~ 80 %–100 % of the total sulfur passes through DMSO, depending on the NO concentration. If we assume that NO is relatively high (e.g., 10 ppb), then the isomerization channel is negligible (~ 1 %–4 % of CH_3_SCH_2_OO fate) (Ye et al., 2022; Assaf et al., 2023), such that all sulfur in the abstraction channel passes through CH_3_S. Under lower-NO conditions, competition with isomerization lowers this fraction to ~ 17 %–41 % (assuming 10 ppt NO and 100 ppt HO_2_ (Ye et al., 2022); isomerization rate = 0.039–0.13 s^−1^ (Ye et al., 2022; Assaf et al., 2023); and bimolecular rates taken from MCM (Saunders et al., 2003)). Based on these assumptions, the addition and abstraction channels can therefore be reasonably represented by DMSO and DMDS chemistry, allowing us to reconstruct DMS aerosol yields using the yields measured in this study and appropriate correction factors based on the literature branching ratios.

**Figure 8.**
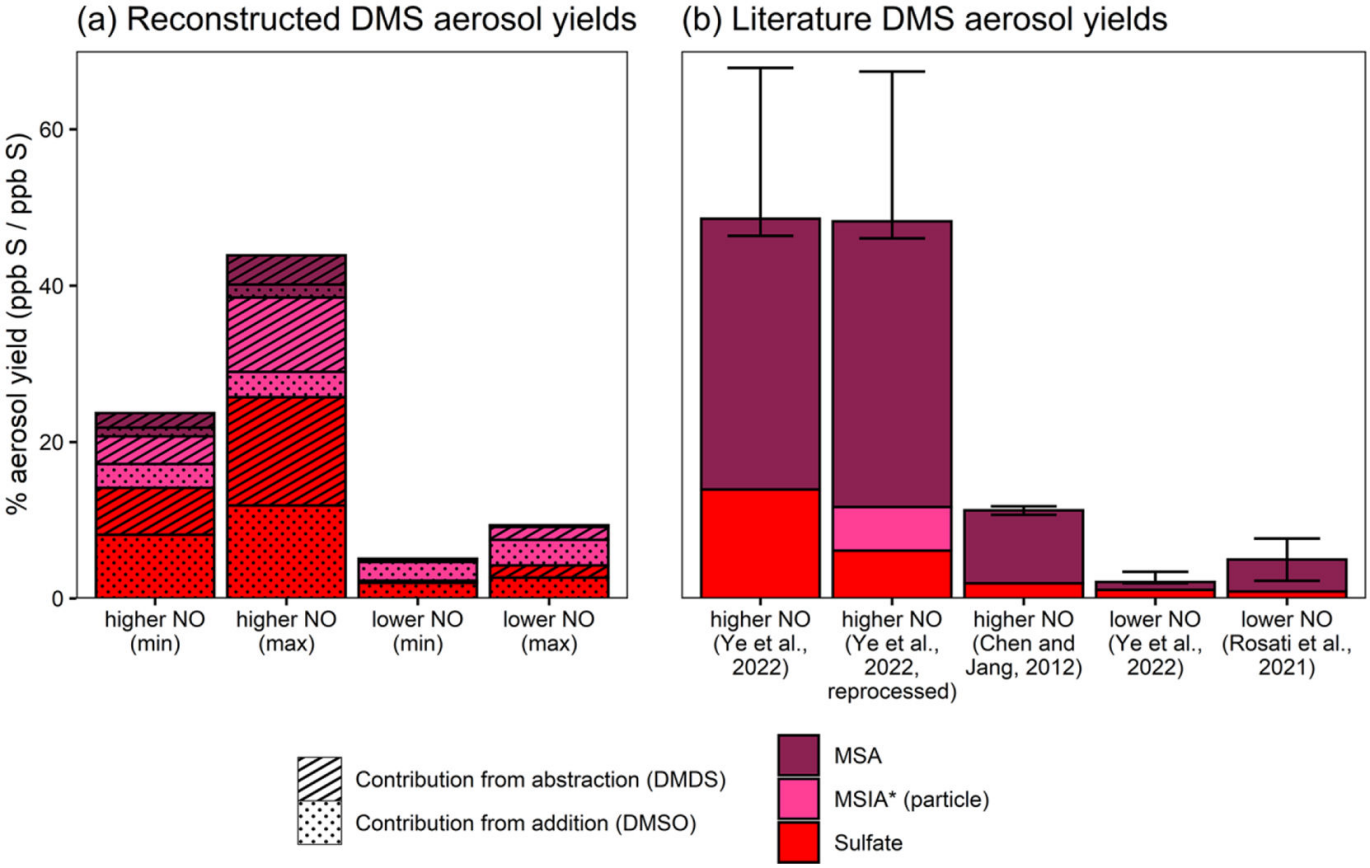
Aerosol yields from DMS as reconstructed from DMSO and DMDS results **(a)** and from the literature measurements (Ye et al., 2022; Chen and Jang, 2012; Rosati et al., 2021) **(b)**. Aerosol yields are shown as product formed divided by DMS reacted (ppb S/ppb S) and only consider rapid aerosol formation. Reconstructed yields shown in the left panel are calculated from DMSO- and DMDS-derived aerosol measurements, as described in the text. In addition to the literature yields, panel **(b)** includes data from Ye et al. (2022), which are reprocessed using the same AMS quantification methods used in this work (see the text and the Supplement for further details).

Figure 8 shows reconstructed DMS aerosol yields from DMSO and DMDS (Fig. 8a) in comparison with the literature DMS aerosol yields (Fig. 8b). These yields only consider rapid aerosol formation and do not include the influence of sulfate formed through SO_2_ oxidation. Reconstructed yields are calculated by multiplying DMSO and DMDS aerosol yields by the appropriate DMS branching fraction for the addition and abstraction channels (36 % and 64 %, respectively). For lower-NO conditions, DMDS aerosol yields are also multiplied by 17 %–41 % to reflect competition with isomerization. For aerosol yields calculated from DMSO, the minimum and maximum values are calculated from the range of yields observed in our experiments. For those calculated from DMDS, the lower bound is based on the total aerosol yield from DMDS, while the upper bound assumes that only 50 % of DMDS sulfur yields CH_3_S and that all aerosol is derived from CH_3_S.

Reconstructed aerosol from DMSO, representing the addition channel, and DMDS, representing the abstraction channel, predicts total DMS aerosol yields of 24 %–44 % at higher NO and 5 %–9 % at lower NO (Fig. 8a). Contributions from the DMSO and DMDS experiments are roughly equal (38 %–88 % from DMSO; 12 %–62 % from DMDS), providing evidence that both abstraction and addition channels represent substantial sources of rapidly formed aerosol.

For comparison, Fig. 8b shows previous measurements of aerosol formation yields from DMS oxidation. At higher NO, reconstructed yields fall slightly below those measured for DMS oxidation by Ye et al. (2022) (experiments performed in the same chamber and under similar conditions at 42–53 ppb NO). However they are substantially greater than values measured by Chen and Jang (2012); those experiments were performed at comparable NO levels (21–117 ppb) but featured higher humidity (28 %–60 %) and did not use seed particles to reduce and account for losses of oxidized products to the chamber walls. At lower NO, reconstructed yields are somewhat greater than those observed in Ye et al. (2022) (~ 10 ppt NO) and roughly consistent with measurements reported by Rosati et al. (2021) (dry chamber; 1–2 ppb background NO_*x*_). While the general trend of higher aerosol yields at higher NO is qualitatively consistent across the reconstructed and literature results, differences in the experimental conditions and wall loss correction methods likely influence the discrepancies in total observed aerosol yields.

While reconstructed yields are largely similar to the measured yields, the differences in composition are substantial. The majority of aerosol from DMS experiments is in the form of MSA (47 %–83 % of total aerosol), while MSA makes up only 2 %–13 % of the total reconstructed yields. The large discrepancy in aerosol composition might be explained by assumptions in the reconstruction of DMS yields. The reconstruction of DMS yields leaves out the possible formation of aerosol from DMSO_2_ or the isomerization pathway. But even if these channels were to form MSA in 100 % yield, their effect on composition under elevated NO conditions would be minor, since they only make up ~ 4 %–7 % total sulfur at 10 ppb NO (Saunders et al., 2003; Burkholder et al., 2020; Ye et al., 2022; Assaf et al., 2023).

Another possible explanation for the discrepancies in composition could be the use of different AMS quantification techniques. When the MSA/MSIA linear combination method from this work is applied to data from Ye et al. (2022), MSIA* is found to be a minor but non-negligible contributor (10 % of total particulate sulfur), while the fraction of MSA actually increases at the expense of sulfate (Fig. 8b; also see the Supplement). This increases the discrepancy between the aerosol composition as measured for DMS and the reconstructed aerosol composition. While the application of this method to older DMS data is imperfect without contemporaneous reference spectra, it demonstrates that it could be a useful technique in field and laboratory studies under conditions where MSA and MSIA are expected to dominate the particle-phase organosulfur composition.

The differences in aerosol composition are most likely due to subtle chemical dependencies that affect branching between SO_2_, MSA, and sulfate. As noted previously, it is possible that high hydrocarbon concentrations in atmospheric chambers relative to the real atmosphere may allow a CH_3_SO_3_ + R–H reaction that increases MSA yields. If the DMS hydrogen is more labile than that of DMSO or DMDS, as is suggested by (somewhat uncertain) OH abstraction rates (Burkholder et al., 2020; González-García et al., 2006), then this may favor MSA production in DMS experiments. The inconsistencies in yield and composition might also be the result of detailed chemistry of simple sulfur radicals (e.g., CH_3_S, CH_3_SO, and CH_3_SO_2_), which could be highly dependent on reaction conditions (e.g., through reactions with HO_2_, NO, NO_2_, and O_3_). Higher relative MSA yields from DMSO seen by Chen and Jang (2012) may, for instance, be influenced by sulfur radical branching caused by the higher-NO concentrations used in that study. While recent work has made important advances in the understanding of these reactions (Chen et al., 2023; Berndt et al., 2023), many remain poorly understood, with mechanisms often relying on basic parameterizations (Saunders et al., 2003) or approximate rate estimates (Yin et al., 1990a); these represent an opportunity for further experimental and computational study.

## 4 Conclusions

In this study, we conducted experiments examining the OH oxidation of DMSO and DMDS. These results are among the first to focus on the amount and composition of aerosol formed from these two compounds and as such identify both agreement with the literature mechanisms and areas where known mechanisms do not describe the observed products. Major products from DMSO oxidation include MSIA, SO_2_, and MSA, and sulfate, while DMSO_2_ is not observed to form. MSA and sulfate yields increase with increasing NO_*x*_, while SO_2_ is observed to form only in the presence of NO_*x*_. These observations, particularly the trend in SO_2_ formation, cannot be fully explained by current mechanisms. While the major MSA and sulfate formation pathways remain somewhat unclear, these results clearly identify DMSO as a precursor of rapid sulfate aerosol formation, in contrast to standard mechanisms for DMSO and MSIA oxidation. We observe rapid sulfate aerosol formation from DMDS oxidation as well, again with a substantial increase in aerosol yield with increasing NO_*x*_. Several S_2_ products are observed for the first time, suggesting that the stabilization of an OH adduct may represent a minor but viable route to further oxidation chemistry.

Based on the overlap with the DMS mechanism (Fig. 1), these results provide insight into the mechanisms of aerosol production from DMS oxidation. While the total aerosol yield can be roughly explained by the upper bound of the combination of DMSO and DMDS results, the previously measured DMS aerosol composition is substantially different, with a much greater MSA component than can be explained by DMSO and DMDS results (Fig. 8). We hypothesize that discrepancies in aerosol composition may be controlled by the chemistry of small sulfur radical intermediates (e.g., CH_3_S, CH_3_SO_2_, and CH_3_SO_3_). This chemistry is poorly constrained, and the reactions of these species under variable chemical conditions (e.g., changing NO, NO_2_, HO_2_, O_3_, or hydrocarbon concentration) represent important targets for future work.

Despite uncertainties in the exact contributions of the addition and abstraction channels to aerosol yield and composition, our results demonstrate that both channels contribute appreciably to rapid aerosol formation from DMS oxidation, especially under elevated NO conditions. While this work highlights necessary changes to DMS oxidation mechanisms, additional laboratory and computational studies that focus on key intermediates and that further explore the influence of environmental parameters (e.g., RH and *T*) are needed in order to develop a mechanism that can fully explain the observed aerosol formation from the oxidation of DMS under the full range of atmospheric conditions.

## Supplementary Material

Supplementary

## Figures and Tables

**Table 1.** Summary of experimental conditions.

Experiment number	Precursor	Precursor conc. (ppb)^a^	Starting oxidant precursor^a^	Perturbation^a^	Perturbation time^b^ (h)
1	DMSO	60	H_2_O_2_ (3 ppm)	HONO (22 ppb), NO (18 ppb)^c^	3.58
2	DMSO	59	HONO (23 ppb), NO (25 ppb)	–	–
3	DMSO	58	H_2_O_2_ (3 ppm)	O_3_ (105 ppb)^d^	2.38
4	DMSO	43	HONO (29 ppb), NO (24 ppb)	–	–
5	DMDS	94	H_2_O_2_ (3 ppm)	NO (22 + 10 ppb)^e^	3.02, 3.20^e^
6	DMDS	61	HONO (16 ppb), NO (11 ppb)	–	–
7	DMDS	97	None^f^	–	–

a Concentrations are reported at *t* = 0 or at the time of perturbation. The concentration of H_2_O_2_ is reported as the total amount added to the chamber. The HONO concentration is measured using the NO_2_ channel of the NO_*x*_ monitor. This represents an upper limit, since [NO_2_] is assumed to be 0 ppb at *t* = 0 (see the Supplement).

b Relative to lights-on time (*t* = 0).

c 600 ppb dichloromethane was also added during this experiment at *t* = 1.92 h but was not observed to affect product formation.

d O_3_ was added to investigate the influence of CH_3_SO_2_ + O_3_ chemistry on product distribution.

e NO was added in two subsequent additions 11 min apart (see Fig. S5; for simplicity, only the time of the first addition is shown in most plots).

f No oxidant precursor added; the experiment measured photolysis only.
