# Supplementary material for: Chamber studies of OH + dimethyl sulfoxide and dimethyl disulfide: insights into the dimethyl sulfide oxidation mechanism
Source: Atmos Chem Phys. Author manuscript; Available in PMC 2024 May 9. (PMC11081431; doi:10.5194/acp-24-1299-2024)
Supplement: Supplementary [file NIHMS1987627-supplement-Supplementary.pdf]

Supplement of Atmos. Chem. Phys., 24, 1299–1314, 2024
https://doi.org/10.5194/acp-24-1299-2024-supplement
© Author(s) 2024. CC BY 4.0 License.

Atmospheric Chemistry and Physics
Open Access

Supplement of

Chamber studies of OH + dimethyl sulfoxide and dimethyl disulfide: insights into the dimethyl sulfide oxidation mechanism

Matthew B. Goss and Jesse H. Kroll

Correspondence to: Matthew B. Goss (mgoss@mit.edu) and Jesse H. Kroll (jhkroll@mit.edu)

The copyright of individual parts of the supplement might differ from the article licence.

Supplement

S.1 Detailed quantification methods for gas-phase species

S.1.1 DMDS

DMDS was quantified using a gas-chromatography-flame-ionization-detector (SRI GC-FID). For each run, air was pulled through a trap (Supelco 2-0370-U) at 0.5 LPM, then heated and flushed onto the column. The full GC method is shown in Table S1.

Table S1: GC-FID operation details showing time, events, and the oven temperature program.

Time (minutes)	Event	Temperature program
0.0	Zero the signal	Hold 50° C for 5 min
0.1	Pump turned on to sample air through trap	
3.0	Pump turned off	
3.1	Trap heater turned on (setpoint = 300 °C)	
4.1	Helium flushed through trap onto column	
5.0		Ramp at 100° C / min to 140° C
5.9		Ramp at 40° C / min to 220° C
7.0	Trap heater turned off	
7.9		Hold at 220° C for 1.1 minutes
8.9	Valves reset to sample position	
9.0	End signal recording	Oven cooling
13.75	Next sample started	

DMDS eluted as a well-defined peak at t = 420-435 seconds. Dichloromethane used to wash the $NaNO_3$ seed particle solution was also detected as a broad peak (t = 350-510 seconds). To separate these, a spline fit to the dichloromethane peak was subtracted from the total signal to yield a clean DMDS signal. The sensitivity of the instrument to DMDS was 8.2 mV s ppb^{-1} based on three independent chamber injections of known volumes and remained stable across experiments performed two months apart.

Due to the long gaps between each measurement timepoint and the short duration of experiment 6, the point at t = 0 for this experiment is extrapolated based on the three points before t = 0 (See Fig. S11b).

S.1.2 Gas-phase organic species

Gas-phase organic species were measured using the NH_4^+-CIMS sampling directly from the chamber (DMDS experiments), or with a mass flow controller supplying clean air to dilute the sample flow by a factor of ~14 (DMSO experiments) to avoid overwhelming the primary ion with the DMSO signal. The dilution factor was quantified by adding acetonitrile to the chamber before the dilution was started; when the dilution flow was started, the reduction in the acetonitrile signal provides a precise measurement of the dilution factor. During experiment 3, the mass flow controller reset to zero, stopping the dilution flow, and overwhelming the primary ion with DMSO. NH_4^+-CIMS data from this experiment are therefore reported with a gap in the timeseries. To facilitate quantification, collision-induced dissociation (CID) scans (Zaytsev et al., 2019) were run during each experiment; timeseries data shown in plots are interpolated across these short gaps.

Raw NH_4^+-CIMS data were processed in PTRwid (Holzinger, 2015). Product ions, detected as mass + NH_4^+, were separated from background ion signals using hierarchical clustering based on their normalized timeseries (Koss et al., 2020). Where analytical standards were not available, instrument sensitivity for each ion was estimated by comparing CID scans to CID scans carried out on a mixture of VOCs of known concentrations (See Zaytsev et al. (2019)). Instrument signal was divided by the sensitivity to obtain concentrations. Calculated sensitivities remained relatively stable between experiments. Compounds for which the signal was too low to show clear response to the CID scan, and compounds whose molecular formula did not represent a closed-shell species were removed.

DMSO was calibrated directly using a liquid calibration unit (Ionicon Analytik). A solution of DMSO (10 μL DMSO in 20 mL MilliQ water) was atomized and vaporized into the NH_4^+-CIMS inlet, and the liquid flow rate was adjusted to provide a multipoint calibration. This was also attempted for MSIA using acidified sodium methanesulfinate but was not successful.

S.1.3 SO_2 and NO_X

The SO_2 monitor (Teledyne T100) and NO_X monitor (Thermo Scientific Model 42i) were calibrated using analytical standard gas cylinders. The quoted detection limits for these instruments are < 0.4 ppb and 0.4 ppb (with 60 second averaging) respectively, however the limit of detection based on the standard deviation of the signals (3σ) when sampling zero air suggest that these limits are slightly below 1 ppb and slightly above 1 ppb respectively. Under low-NO_X conditions (H_2O_2 + UV + VOC) after several days of flushing with clean air, the total NO levels in our chamber have previously been estimated to be around 10 ppt (Ye et al., 2022), based on the changing yield of an organonitrate species measured with the NH_4^+-CIMS before and after NO addition.

The NO_X monitor measures NO, total NO_X, and by subtraction, NO_2. For the total NO_X channel, NO_2 and other NO_Y species are catalytically converted to NO, such that NO_2 and HONO are indistinguishable. Assuming unit conversion of NO_Y species, the NO_2 channel therefore represents NO_2 + NO_Y. For experiments where HONO is added, the entirety of the NO_2 signal at the beginning of the experiment is assumed to be HONO (Table 1) though this represents an

upper bound. Substantial NO is also observed to form when HONO is generated (see Fig. S4 and S5).

S.1.4 Data synthesis

All gas-phase species were corrected for dilution loss by dividing by a normalized exponential fit of the acetonitrile timeseries. This fit is derived from t = 0 to the end of the experiment, except for experiment 5, for which the fit is carried from t = 1500 s to the end of the experiment due to a deviation in the acetonitrile timeseries. Gas-phase and particle-phase data are combined into one dataset, background subtracted, and block averaged on 180 second intervals for most plots.

S.2 Detailed AMS quantification methods

Quantification of particle-phase products using the AMS followed a new method developed to measure the concentration of MSIA and MSA. Prior to the experiments, the AMS relative ionization efficiency (RIE) for MSA was calibrated following the method of Hodshire et al. (2019). Methanesulfonic acid (Signa Aldrich, > 99.0%) was reacted with excess ammonium hydroxide (VWR, 28-30%) to form a solution of ammonium methanesulfonate (NH_4MSA). Reference spectra were also taken for ammonium sulfate (Sigma Aldrich, >99.0%), ammonium methanesulfonate, and sodium methanesulfinate (NaMSIA) (Sigma Aldrich, 85%) by atomizing these compounds into the AMS. These spectra are processed as described below and shown in Fig. S1.

To process experimental data, raw AMS data is first processed in Igor. AMS data were processed in SQUIRREL 1.63B and PIKA 1.23B to obtain high resolution product spectra. These data are grouped by chemical family and exported as matrices of spectra over time. This is done for HRSO4, HRNO3, familyCS, familyOther, familyCH, familyCHO1, familyCHOgt1, and familyCx.

Next, the exported data are processed in R to reshape the data structure. The exported matrices are first recalculated to be timeseries over each unit-mass-resolution m/z value instead of each HR ion. Only peaks below *m/z* 115 are used due to uncertainty in fit for higher masses. Timeseries corresponding to HS^+, H_2S^+, H_3S^+, and their isotopes are taken from familyOther and added to familyCS. All C-containing families (CH, CHO1, CHOgt1, and Cx) are summed together to make a new familyC. Note that “HR aerosol species” have the HR Frag Table applied, which corrects for specific overlapping peaks, but “HR families” do not. This matters most notably for S^+ which overlaps with the O_2^+ signal. Using “HRSO4” (the aerosol species) corrects for this issue. Therefore, the new familyCS, familyC, and HRSO4 data are saved for further analysis. This process of restructuring HR output data is also done for the reference spectra. However, for the reference spectra, any non-sulfur-containing organic peak with a mass greater than CH_4O^+ is set to zero to avoid including any background ions in the reference. These reference spectra are shown in Fig. S1.

The reference spectra demonstrate the differences between MSA and MSIA as they appear in the AMS. While they share similar dominant peaks, the MSA spectrum features notable familyCS peaks at masses 78 ($CH_2SO_2^+$), 79 ($CH_3SO_2^+$), and 96 ($CH_4SO_3^+$). The distribution of familyCS

peaks with masses below m/z 63 is also different. While MSA is usually quantified based on the m/z 79 peak (Hodshire et al., 2019), we observe a small signal from MSIA at the same mass, suggesting that this cannot uniquely separate them. We therefore rely on a linear combination of the MSA and MSIA spectra to derive their respective components from the product spectra. We note that we observe a peak at m/z 125 in the MSIA reference spectrum that is not able to be identified with this mass spectral resolution. This may be due to an impurity in the technical grade NaMSIA standard and due to the uncertainty in its identification, it is left out of further analysis.

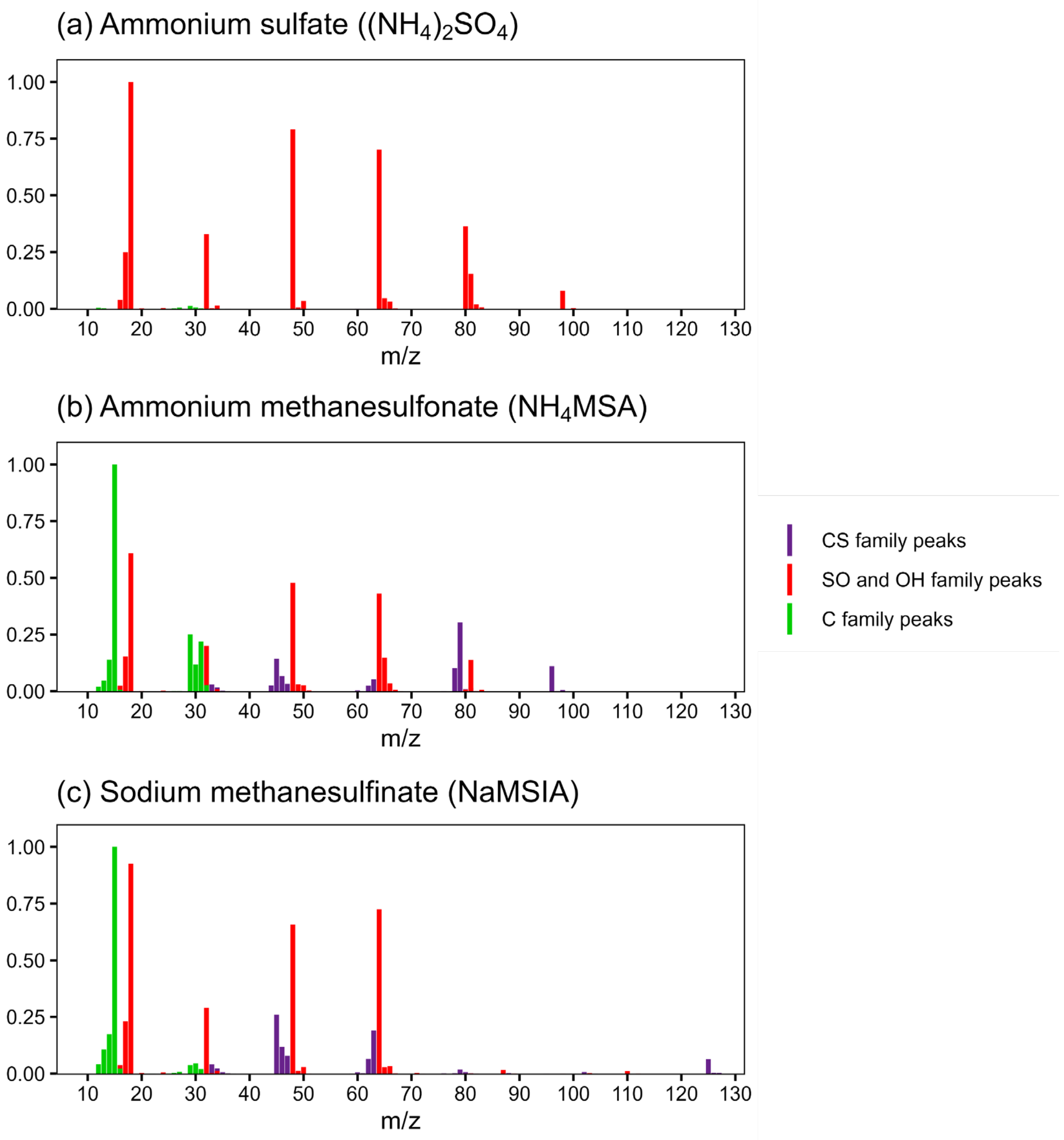

Figure S1: Reference spectra for ammonium sulfate (Panel a), ammonium methanesulfonate (Panel b), and sodium methanesulfinate (Panel c). These spectra leave out the ammonium peaks for simplicity and focus only on the chemical families used in this analysis. Chemical families are grouped as described in the text. "SO and OH family peaks" refers to the "HRSO4" family generated in PIKA.

Following this reclassification of some species, the data are fit as a linear combination of reference spectra to derive MSIA and MSA factors. Since the most unique features of the mass spectra for MSA and MSIA are peaks containing C, H, and S, only familyCS peaks are used for the linear fit. At each timepoint, the familyCS spectrum from the experimental AMS data is fit as a linear combination of the familyCS spectra from the MSA and MSIA reference data with 0 set as the intercept. These fit coefficients are then applied to the full reference MSA and MSIA spectra, and the peaks are summed to generate a timeseries of MSA and MSIA. The r^2 value from the linear combination was typically around 0.95 in these experiments. An example timeseries of the MSA and MSIA coefficients from the regression and the r^2 value are shown in Fig. S2.

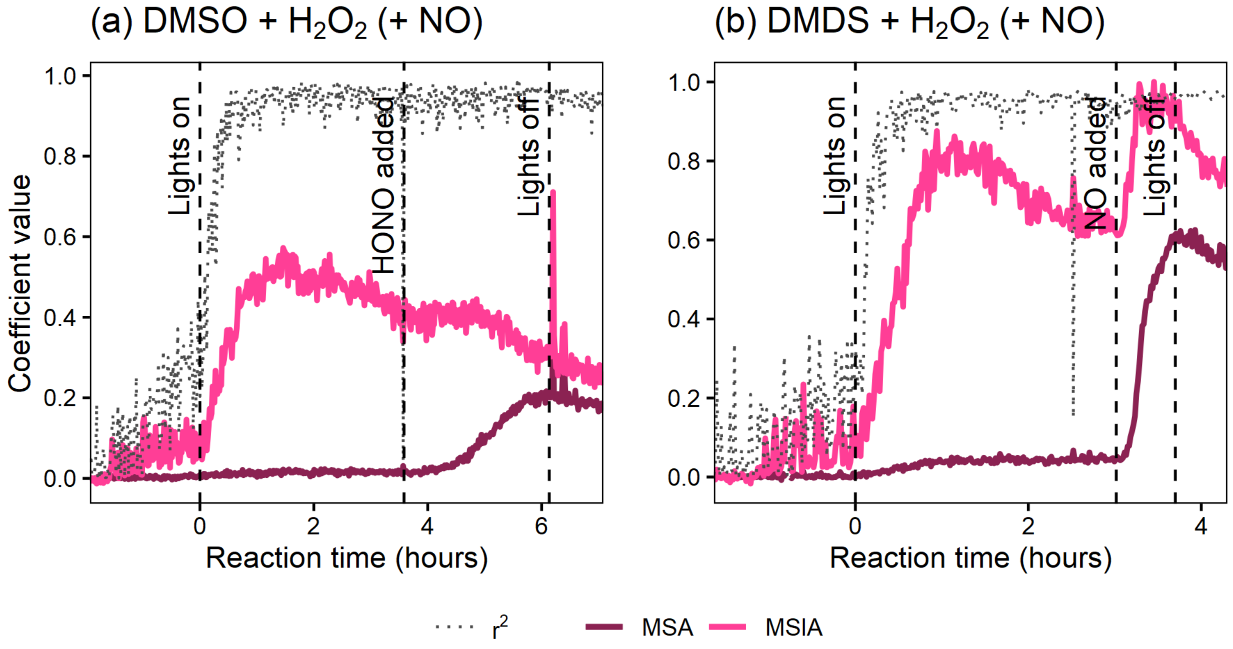

Figure S2: Example timeseries of the MSA and MSIA coefficients from the linear combination fits of familyCS peaks, in addition to the r^2 values over time. Panel a shows the fit from experiment 1 in which DMSO was oxidized (median r^2 = 0.95). Panel b shows the fit from experiment 5 in which DMDS was oxidized (median r^2 = 0.96).

To generate a sulfate timeseries, the "HRSO4" peaks from the derived MSA and MSIA spectral timeseries are subtracted from the original HRSO4 spectra over time. This gives a residual sulfate spectrum over time. The peaks are summed to generate a sulfate timeseries. We note that the distribution of sulfate peaks is not constant over time and appears to be influenced by the sodium counterion. $NaSO_3^+$ is first seen in much greater abundance than SO_3^+ but these ions swap in relative abundance as the experiment proceeds. This is likely due to loss of $NaNO_3$ seed particles to the walls and dilution while sulfate continues to be produced through gas-phase chemistry. An example of this change over time is shown in Fig. S3.

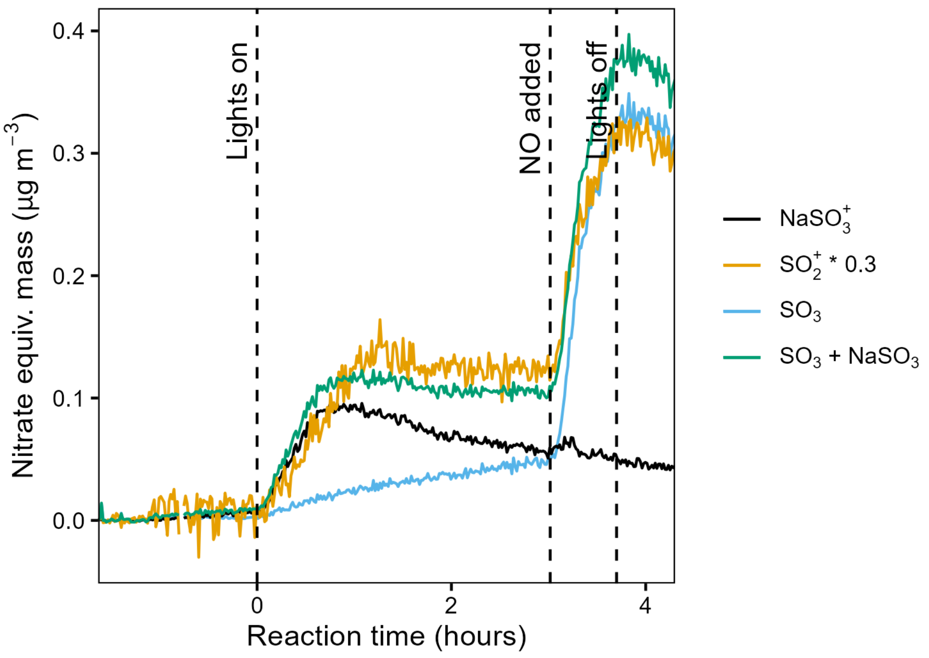

Figure S3: Timeseries of selected AMS ions from experiment 5 (DMDS). The spectrum of the sulfate residual shifts over time, most dramatically between the SO_3^+ and the $NaSO_3^+$ peaks. As sulfate is produced throughout the experiment, sulfuric acid becomes the more dominant form, shifting SO_3 away from the Na^+ counterion. Summing the SO_3^+ and $NaSO_3^+$ signal gives a timeseries that is relatively consistent with the SO_2^+ signal, suggesting that this is merely a change in counterion rather than a different sulfate species. After these ions are summed, the full spectrum is relatively constant over the length of the experiment.

The same subtraction approach is used to generate an organic residual timeseries. This is small (generally 1-2 µg m^{-3} before dilution and wall loss correction) and is introduced as an organic contaminant on the seed aerosol particles. It generally decreases with the seed aerosol signal though in some cases increases slightly towards the end of experiments. Since it does not contain sulfur, it is not considered further in this analysis.

Since the derived timeseries are in nitrate equivalent mass, appropriate RIE values are applied to each species. We measured RIE for MSA to be 2.06 using the ammonium balance method (Hodshire et al., 2019). Since we do not have ammonium methanesulfinate (NH_4MSIA), this method cannot be used for MSIA and we therefore assume the same RIE value for MSIA. Data are wall loss and dilution corrected by dividing by the normalized HRNO3 timeseries, and collection efficiency is corrected for by comparing the mass of the seed particles measured by the AMS to SMPS data. The timeseries are finally converted to ppbS based on literature molecular weights and densities for MSA and MSIA.

We note that deconvolving AMS spectra involves inherent uncertainty due to the nature of fragmentation observed (for example, organic species are typically assigned to a general “Org” signal due to the number and complexity of common peaks). For systems such as DMSO oxidation where MSA and MSIA are the only likely organosulfur species that can contribute to the aerosol, this decomposition appears to work well. As noted in the main text, DMDS

oxidation generates additional organosulfur species which may condense onto the aerosol particles. If found in the aerosol phase, these may fragment to form similar distributions of common organosulfur ions (e.g. CHS^+, CH_2S^+ etc.). As possible evidence of these larger species, we observe a small peak at m/z 141 for DMDS oxidation experiments, likely similar to that seen by Van Rooy et al. (2021a), but we were unable to conclusively identify it. While the decomposition between MSA and MSIA fractions represents a reasonable best estimate based on expected products and the reference standards available and consistently fits the data well (see Fig. S2), it may not describe the actual species present in the aerosol, particularly for complex organosulfur oxidation systems. The discrepancy in apparent gas-aerosol partitioning for MSIA in DMSO vs DMDS data is described in the main text, and used to conclude that the MSIA fraction may consist of additional organosulfur species. Given this uncertainty, MSIA concentrations derived from AMS measurements are labeled MSIA*. This method should therefore be used to analyze data for which both MSA and MSIA are expected to be present and should be accorded appropriate uncertainty where other organosulfur species may significantly contribute to the aerosol.

S.3 NO_X and Ozone data

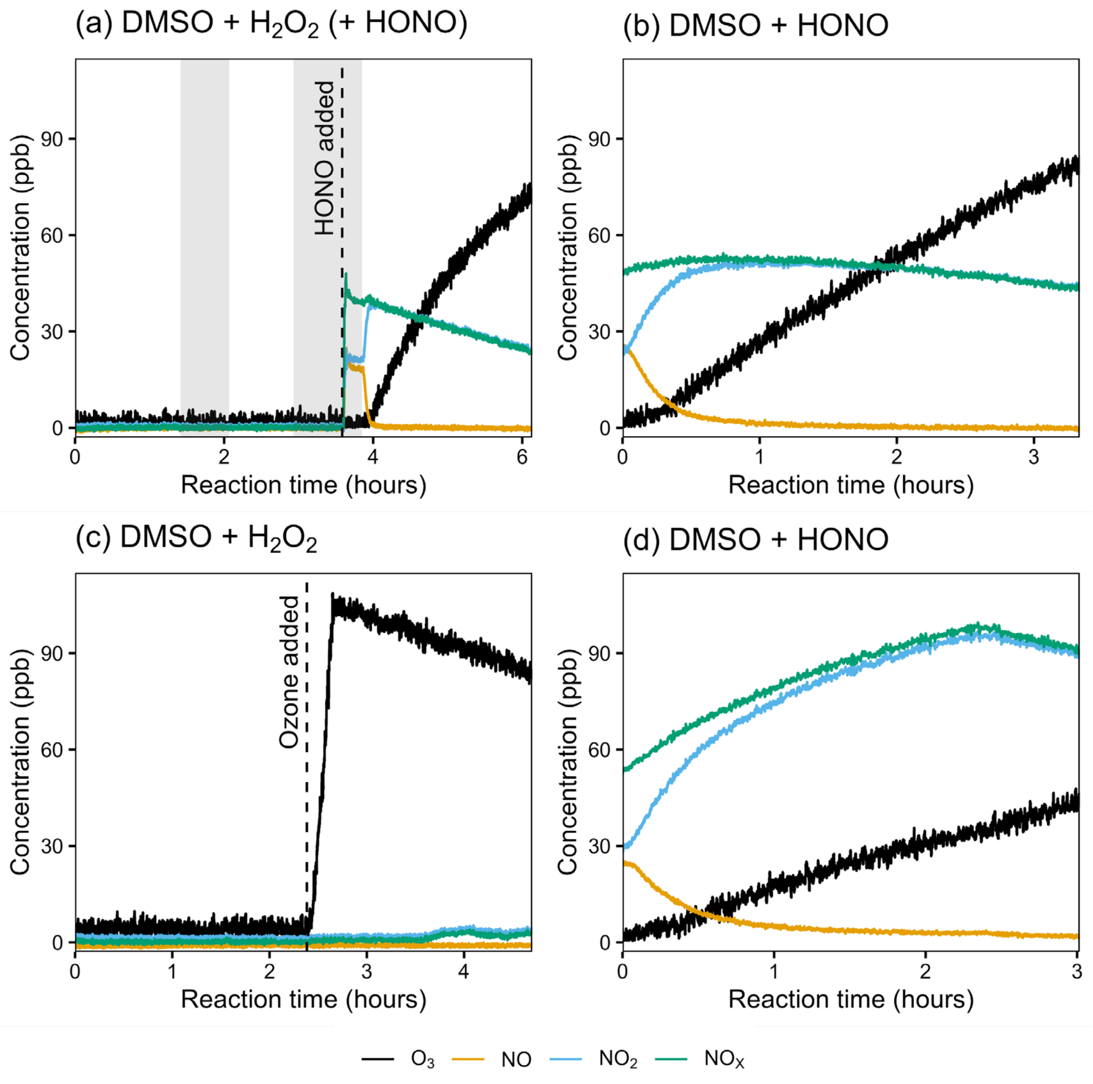

Figure S4: NO_X and ozone timeseries from DMSO oxidation experiments (Panels a-d correspond to experiments 1-4 respectively). Note that NO_Y species such as HONO are measured as NO_2 in the NO_X monitor so "NO_2" represents NO_2 + NO_Y. No dilution corrections have been applied to these data. Note that the x-axes are different in each panel. The gray bars represent times when the lights were turned off for diagnostic purposes.

Figure S4 shows NO_X and ozone timeseries from experiments in which DMSO was oxidized. As described briefly in the main text, HONO (detected as NO_2) is formed with considerable NO as a byproduct (Panels a, b, d). Total NO_X is observed to rise in Panels b and d; this is due to passive diffusion of HONO from the headspace of the H_2SO_4 and $NaNO_2$ solution through an open line to the chamber. In experiment 4, this line was left open until it was fully disconnected at t = 2.37 hours. The HONO solution was also left connected to the chamber for experiment 2 but appears to have diffused more slowly.

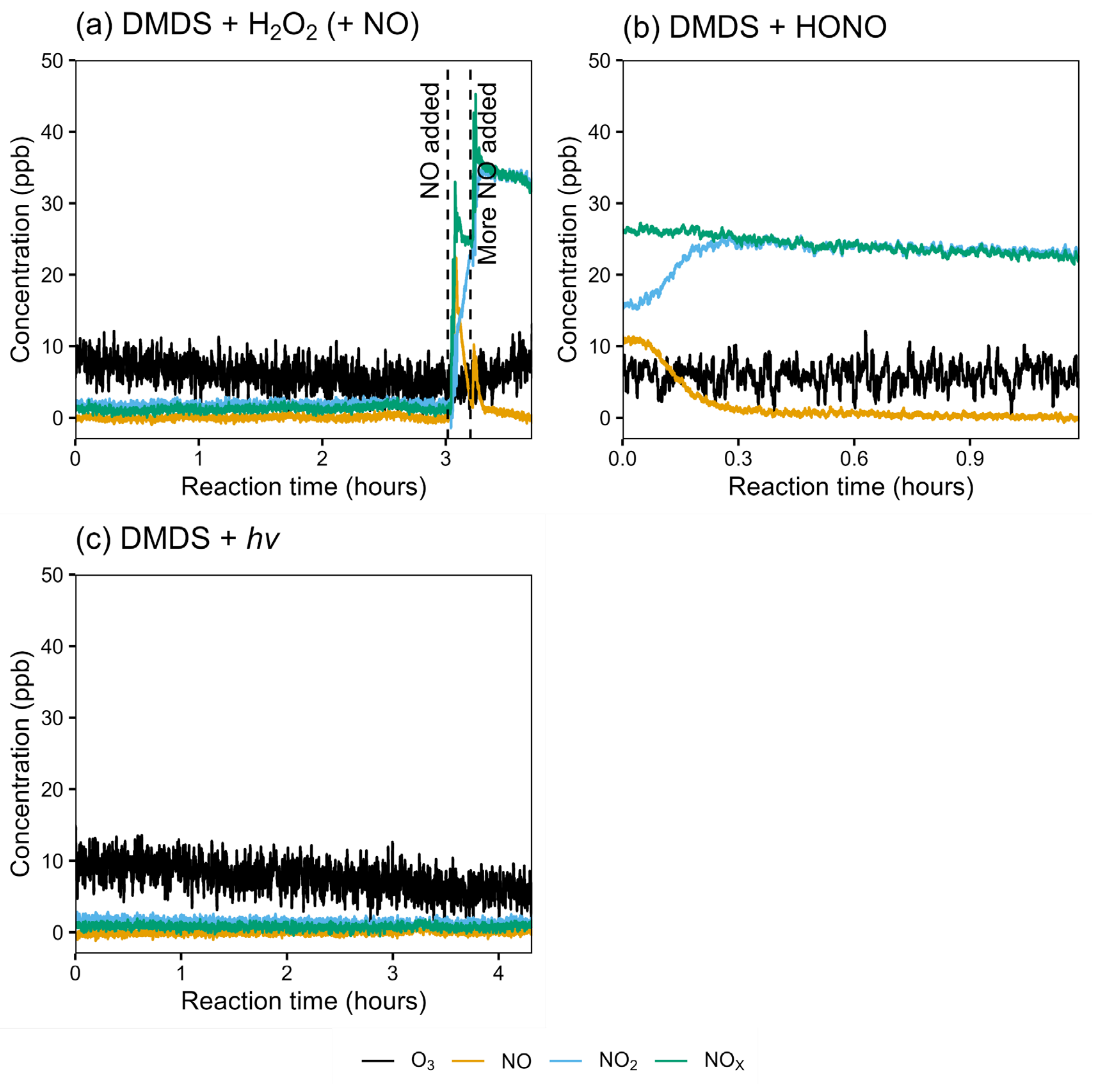

Figure S5: NO_X and ozone timeseries from DMDS oxidation experiments (Panels a-c correspond to experiments 5-7 respectively). Note that NO_Y species such as HONO are measured as NO_2 in the NO_X monitor so "NO_2" represents $NO_2 + NO_Y$. No dilution corrections have been applied to these data. Note that the x-axes are different in each panel.

Figure S5 shows NO_X and ozone timeseries from experiments in which DMDS was oxidized. Most notably, apparent ozone formation is considerably diminished compared to DMSO experiments, possibly due to several factors. The use of only 50% UV light intensity for experiments 5 and 6 should reduce the rate of NO_2 photolysis and therefore O_3 production by 50%. DMDS also interferes with the ozone measurement due to relatively strong absorption at the same wavelength used by the ozone monitor. This cross sensitivity is responsible for the nonzero O_3 concentrations at $t = 0$ in each experiment. Since detection of DMDS in the ozone monitor requires that it be removed by the ozone scrubber in the instrument, its absorption on the scrubber may represent contamination that reduces the sensitivity of the instrument to ozone. The continued diffusion of HONO into the chamber that was seen for DMSO appears to have been negligible for experiment 6.

S.4 Total sulfur plots

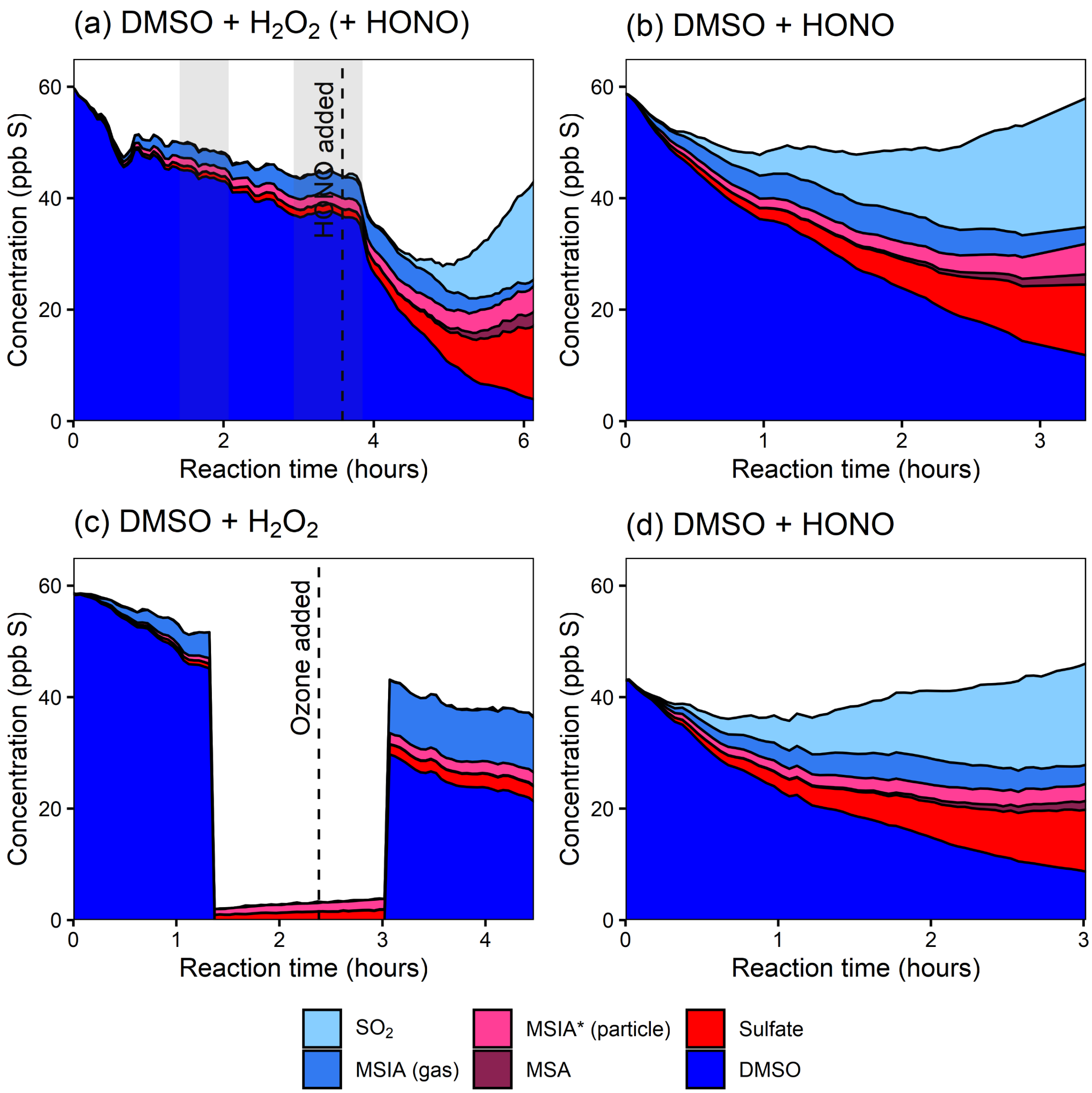

Figure S6: Stacked DMSO and product timeseries for the oxidation of DMSO with H_2O_2 (+ HONO addition) (experiment 1, Panel a), HONO (experiment 2, Panel b), H_2O_2 (experiment 3, Panel c), and HONO (experiment 4, Panel d) as OH precursors. The DMSO signal is somewhat unstable (esp. in experiment 1), likely representing a substantial contribution to the uncertainty. Sulfur closure is variable but suggests that most products are measured within uncertainty. Gray bars denote time periods when the lights were turned off for diagnostic purposes.

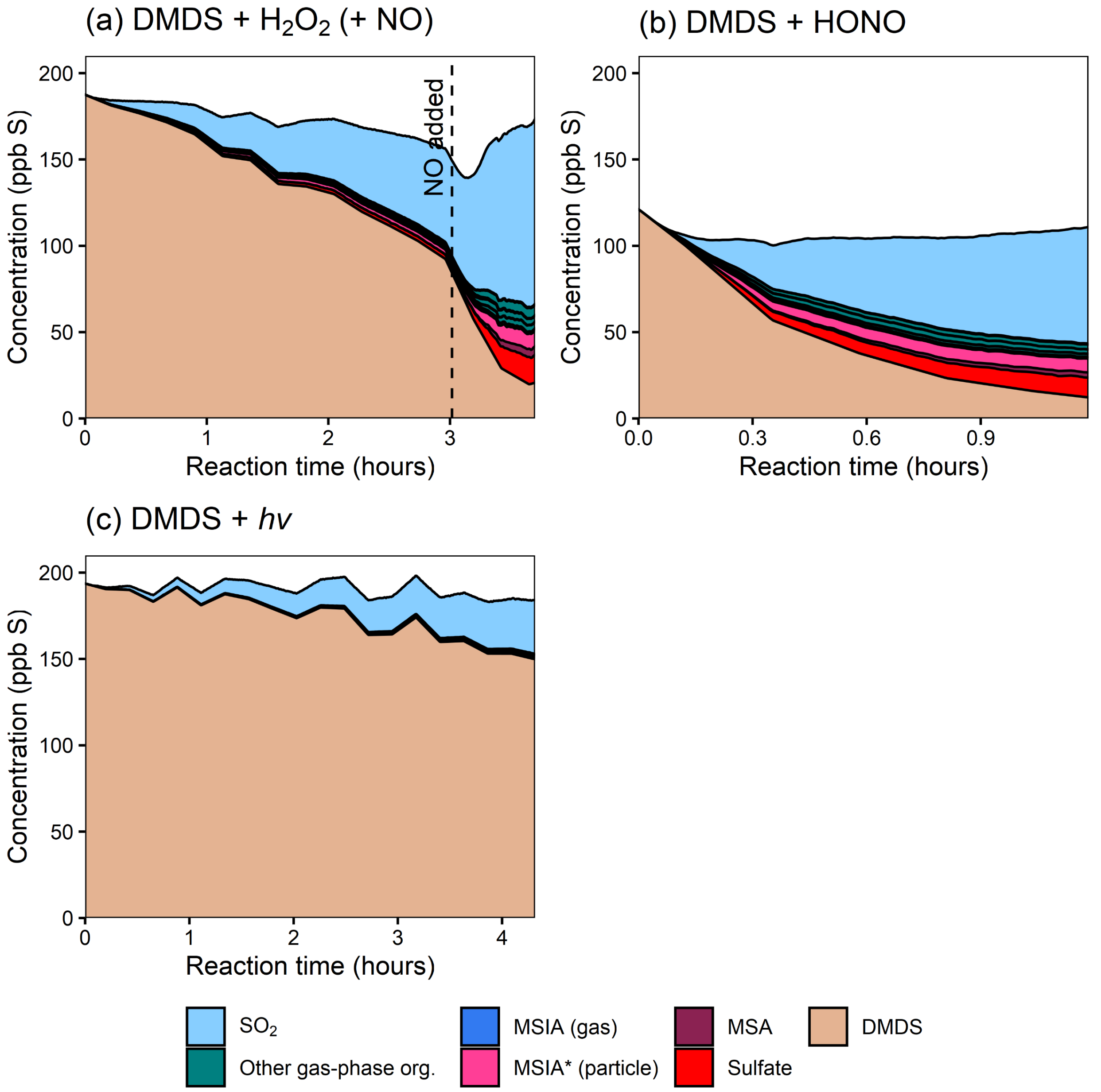

Figure S7: Stacked DMDS and product timeseries for the oxidation of DMDS with H_2O_2 (+ NO addition) (experiment 5, Panel a), and HONO (experiment 6, Panel b) as OH precursors, and the photolysis of DMDS (experiment 7, Panel c). Note that DMDS measurements are taken via GC-FID only every 13.75 minutes. The total sulfur measured remains relatively constant throughout each experiment, suggesting that no major products are unmeasured.

S.5 Product stack plots for additional experiments

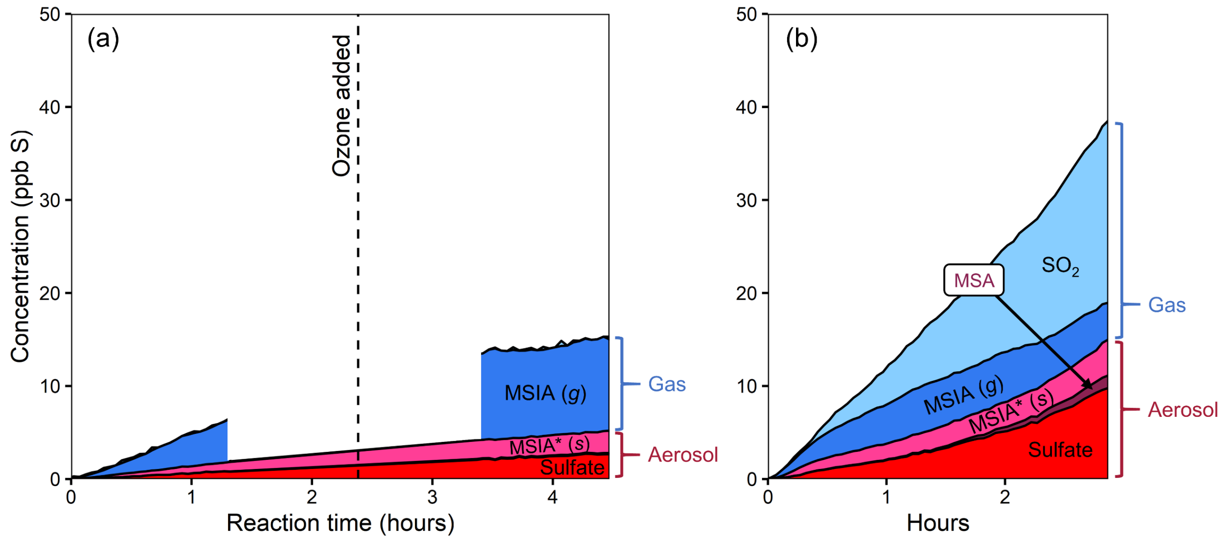

Figure S8: Stacked product timeseries from the oxidation of DMSO with H_2O_2 (experiment 3, Panel a) and HONO (experiment 4, Panel b) as oxidant precursors. The gap in the MSIA (*g*) timeseries for Panel a is due to a malfunction in dilution system in front of the NH_4^+-CIMS inlet.

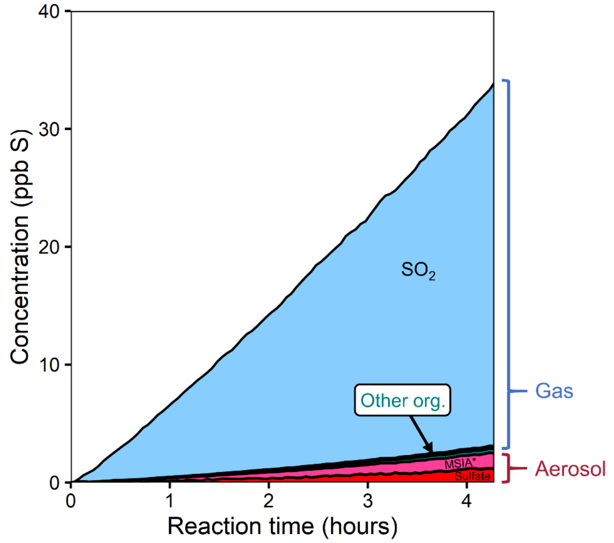

Figure S9: Stacked product timeseries from the photolysis of DMDS with no added oxidant precursor (experiment 7). All gas-phase organic compounds detected by the NH_4^+-CIMS except for MSIA (*g*) are shown in green as "Other org." and shown in greater detail in Fig. S14. Particle phase MSIA* is marked with an asterisk to denote uncertainty in its exact chemical speciation.

S.6 Precursor signal smoothing

For both DMSO and DMDS, the precursor timeseries exhibit moderate noise for some experiments. Where this noise is substantial, non-monotonic decreases in the raw precursor signal would result in discontinuous yield curves (Figs. 3 and 6) when plotted using the "precursor lost" as an x-axis. To render the yield plots more easily interpretable, precursor timeseries are smoothed using penalized splines before a "precursor lost" vector is generated. These smoothed timeseries are shown in Figs. S10 and S11. The DMSO signal ($C_2H_6SO(NH_4^+)$) was less stable than the signals of other ions detected by the NH_4^+-CIMS, particularly for experiment 1 (Fig. S10a), suggesting that the DMSO concentration in the chamber may be somewhat uncertain.

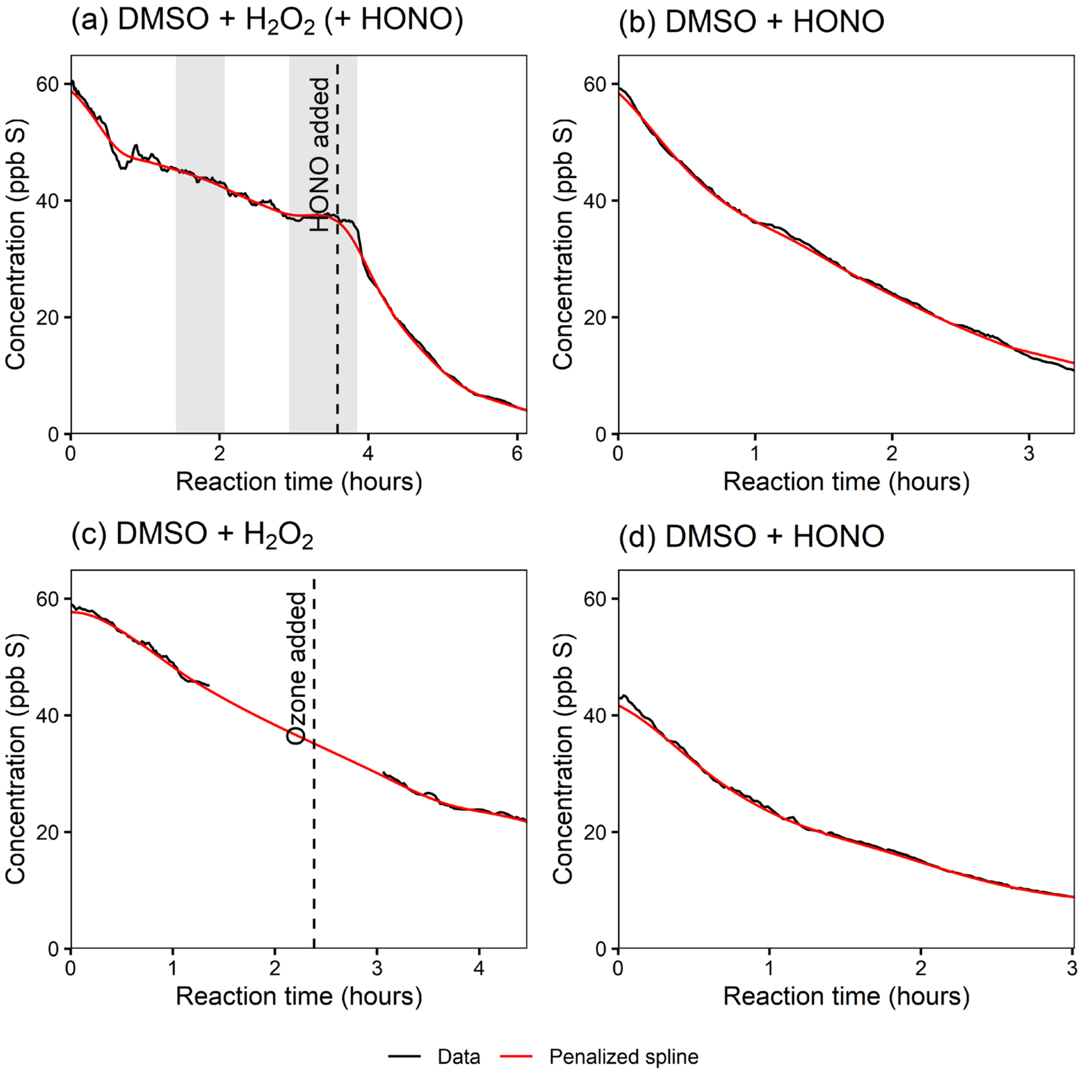

Figure S10: DMSO signal and penalized spline fits used for x-axes of the yield plots (Fig. 3). Panels a-d show data from experiments 1-4 respectively. The splines are fit using the "sm.spline" function from the "pspline" package in R, with spar values set to 1 x 10^9. Gray bars denote times when the lights were turned off for diagnostic purposes.

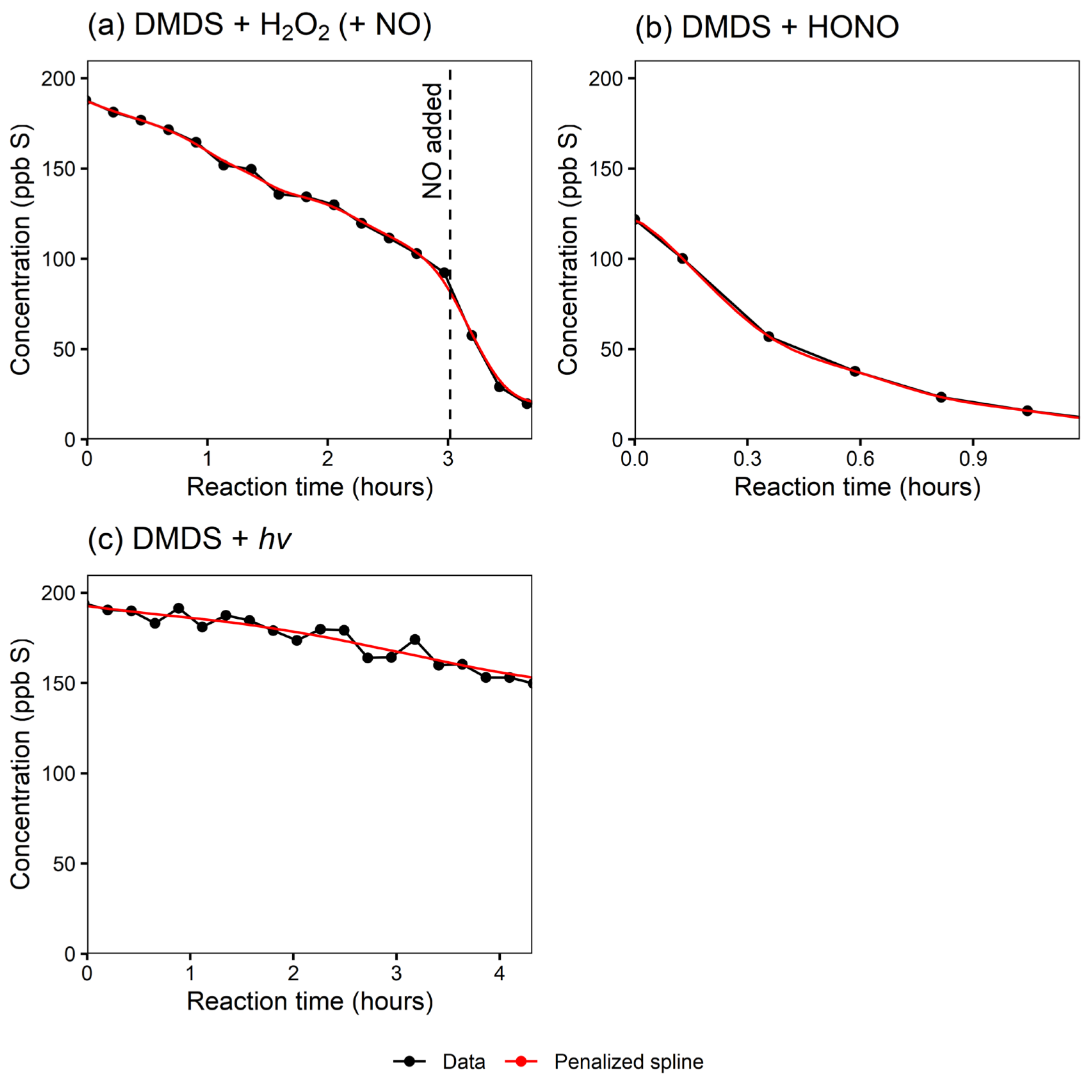

Figure S11: DMDS signal and penalized spline fits used for x-axes of the yield plots (Fig. 6). Panels a-c show data from experiments 5-7 respectively. The splines are fit using the "sm.spline" function from the "pspline" package in R, with spar values set to 1 x 10^8, 1 x 10^4, and 1 x 10^{10} respectively. The point at t = 0 for experiment 6 is extrapolated based on the three points before t = 0.

S.7 Additional plots related to product yield curves

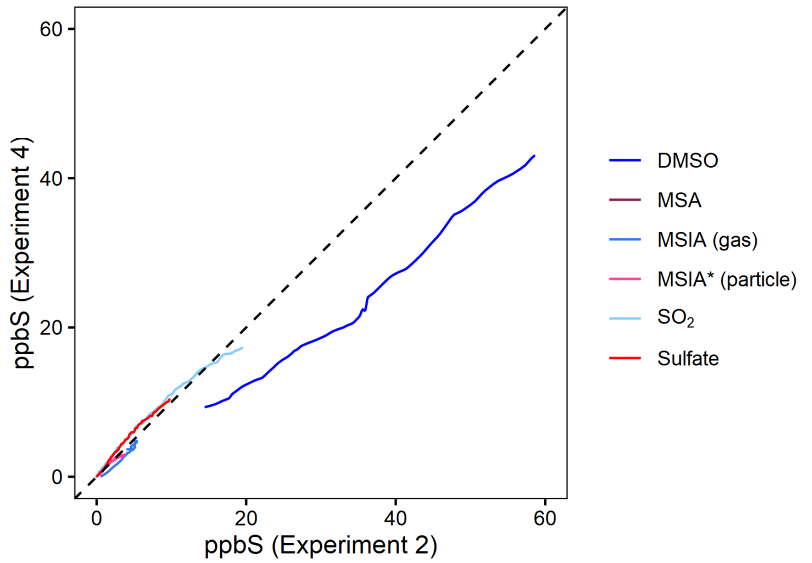

Figure S12: Comparison of replicate DMSO + HONO experiments. The timeseries of each species from Experiment 2 (DMSO + HONO) is plotted against its complement from Experiment 4 (DMSO + HONO replicate). All species fall near the 1:1 line, with the exception of DMSO. This suggests that the majority of the discrepancies between these two experiments as presented in Figure 3 are due to the discrepancy in the measured DMSO concentration.

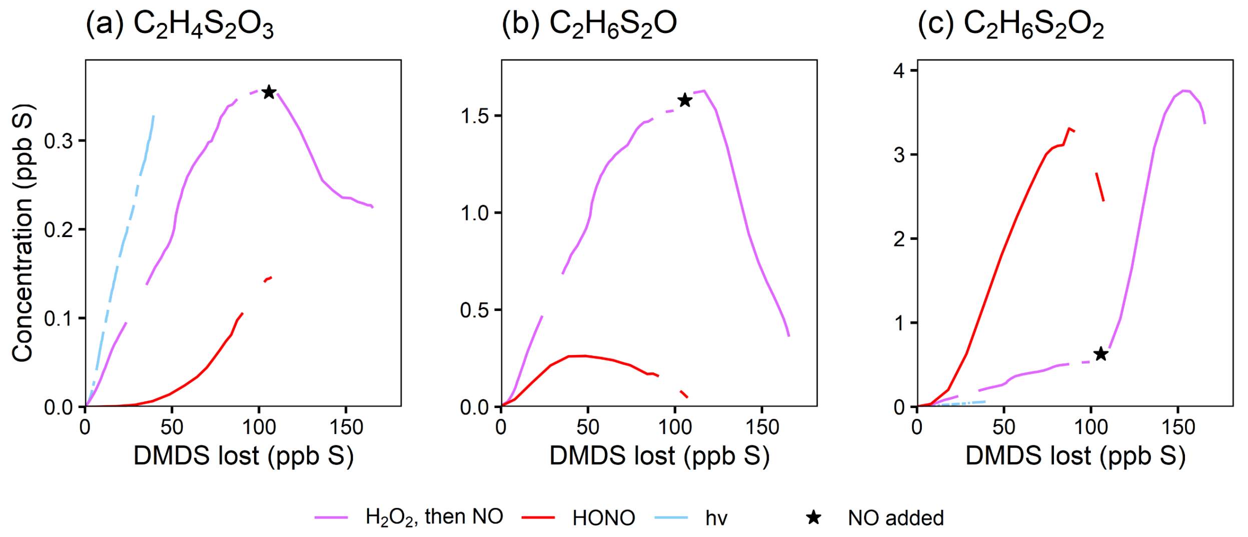

Figure S13: Additional yield plots for selected DMDS oxidation products. $C_2H_4S_2O_3$, $C_2H_6S_2O$, and $C_2H_6S_2O_2$ are plotted vs the loss of DMDS to normalize for changing OH concentrations, allowing comparisons between experiments 5-7. Colors denote experimental conditions. Note that for one experiment, the NO_X regime is switched by adding NO, as marked by the star. Note the differing y axes.

Figure S13 shows yield plots for $C_2H_4S_2O_3$, $C_2H_6S_2O$, and $C_2H_6S_2O_2$. The $C_2H_4S_2O_3$ signal is attributed to the isomerization product $HOOCH_2SSCHO$ (Fig. 1; Fig. S13a). This product is thought to be formed through abstraction of a methyl hydrogen from DMDS, followed by two isomerization steps, and is analogous to the formation of HPMTF from DMS (Berndt et al., 2020). The yield of this product shifts with experimental conditions in response to the RO_2 bimolecular lifetime. Photolysis conditions generate $C_2H_4S_2O_3$ in the highest yield, indicating that some OH is likely present and that alternative RO_2 sinks (HO_2 and NO) are quite low. Experiments using H_2O_2 and HONO as OH precursors (expts 5 and 6) involve shorter bimolecular lifetimes and lower $C_2H_4S_2O_3$ yields. Figures S13b and S13c are discussed in the following section.

S.8 Further discussion of NH_4^+-CIMS data

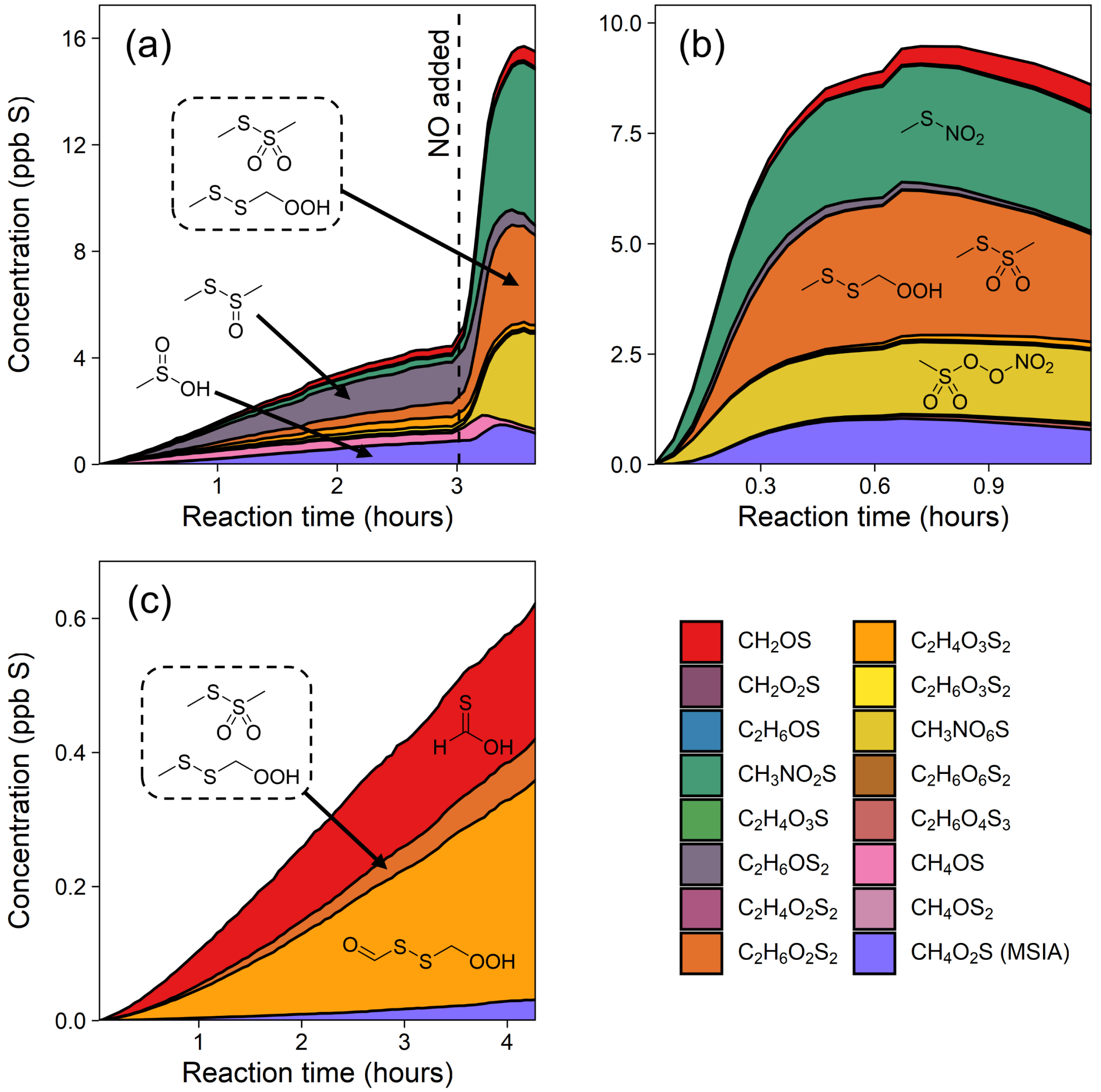

Figure S14: Stacked timeseries of minor gas-phase organosulfur products detected by the NH_4^+-CIMS for experiments 5-7 (Panels a-c respectively). Hypothesized structures for the most abundant products are given. The legend lists the formulas for all closed-shell species detected in the NH_4^+-CIMS. Note that the y-axis is different in each plot. Note that these plots include MSIA which is detected in the NH_4^+-CIMS but not included in "Other org." as shown in Figs. 5 and 7 in the main text.

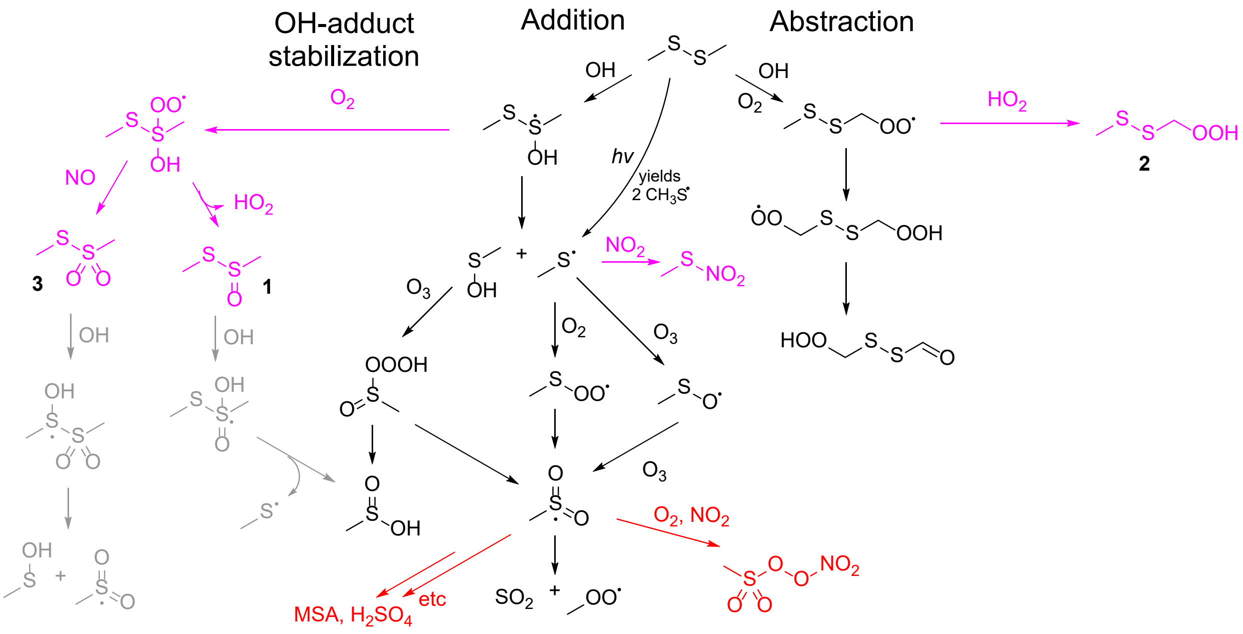

Figure S15: Detailed DMDS oxidation chemistry. Reactions shown in black are taken from Berndt et al. (2020). Reactions shown in red explain observed products based on known DMS chemistry (Saunders et al., 2003). Reactions shown in pink represent new hypothesized reaction pathways. Reactions in gray represent hypothesized secondary chemistry. Several compounds are numbered for reference in the text.

Figures S14 shows the timeseries of all gas-phase organics detected by the NH_4^+-CIMS and Figure S15 shows a detailed DMDS mechanism, based on these results. As discussed in the main text, $C_2H_6S_2O$ forms in greater yield under lower-NO conditions and is best explained by the structure $CH_3SS(O)CH_3$ (product **1** in Fig. S15; see also Fig. S13b). In contrast, $C_2H_6S_2O_2$ forms in greater yield under higher-NO conditions (Fig. S13c). This signal may be from CH_3SSCH_2OOH (product **2** in Fig. S15) at lower [NO], but it is better explained by $CH_3SS(O)_2CH_3$ under higher-NO conditions (product **3** in Fig. S15). Additional oxidation via OH may lead to fragmentation (shown in gray in Fig. S15), resulting in the formation of established DMDS products.

While Van Rooy et al. (2021a) lacked direct observational evidence of $CH_3SS(O)CH_3$ (product **1**), they hypothesized that this molecule might explain peaks in their AMS spectra and suggested that it may form via a condensation reaction between two CH_3SOH molecules. The gas-phase reaction of two closed-shell molecules is likely to be slow and does not explain the observed trends in $C_2H_6S_2O_2$ formation.

Section S.9 Box modeling

All box-modeling is done using the Framework for 0-Dimensional Atmospheric Modeling (F0AM) (Wolfe et al., 2016) and initialization conditions taken from Table 1 in the main text. Chamber light conditions and other model set-up parameters are the same as those used in Ye et al. (2022). All rates are taken from the Master Chemical Mechanism (MCMv3.3.1) unless otherwise noted (Saunders et al., 2003). Additional rates for three hypothesized mechanisms are included in Table S2.

Table S2: Reaction rates for possible DMSO oxidation pathways

Reaction number	Reaction	Rate (cm^3 $molec.^{-1}$ s^{-1})	Citation
	CH_3SO_3 channel		
1	MSIA + OH → CH_3SO_2 + H_2O	9×10^{-11}	(Kukui et al., 2003)
1b	*Remove* MSIA + OH → SO_2 + CH_3 + H_2O		
	OH abstraction[a]		
2[b,c]	MSIA + OH → HOS(O)CH_2OO + H_2O	2.1×10^{-12}	(González-García et al., 2006)
3[d]	HOS(O)CH_2OO + NO → SO_2 + HCHO + HO_2	$2.7 \times 10^{-12} \times exp(360 / T)$	(Saunders et al., 2003)
	OH addition[a]		
4[c,e]	MSIA + OH → $(HO)_2S(O)CH_3$ + O_2 → MSA + HO_2	2.64×10^{-10}	(González-García et al., 2007)

[a] Note that the reactions from the CH_3SO_3 channel have been included in all schemes except the default MCM mechanism.

[b] Uses calculated DMSO + OH abstraction rate for the methyl H as a best estimate. The MSIA + OH abstraction rate for the methyl H has not been calculated.

[c] Rates taken from González-García et al. (2006, 2007) correspond to 298 K.

[d] Generic MCM RO_2 + NO reaction rate.

[e] Note that this computationally-derived rate exceeds the total measured MSIA + OH rate (Kukui et al., 2003). While González-García et al. (2007) calculate the rate for OH addition, they do not consider the possible subsequent reaction with O_2 to form MSA. Here, we assume that reaction with OH is the rate limiting step and reaction with O_2 is fast. While this rate is likely unrealistically fast, to our knowledge no other estimates have been made.

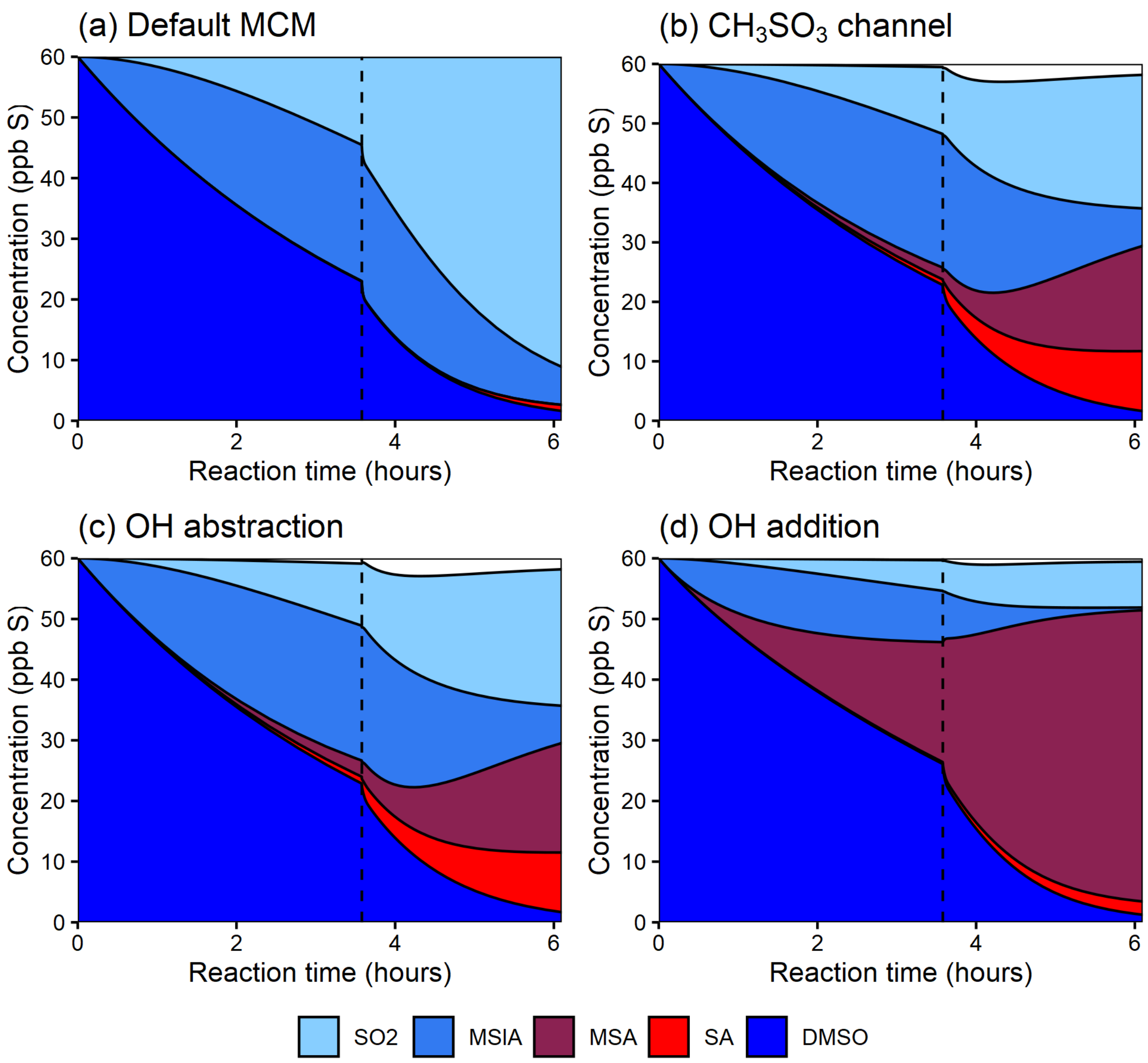

Figure S16: Stacked timeseries of major species for model runs representing experiment 1 using four chemical mechanisms (Table S2). The dashed lines indicate the time at which the HONO/NO perturbation was added. Panels include: (a) default MCM/JPL mechanism, (b) CH_3SO_3 channel, (c) OH abstraction, and (d) OH addition.

Figure S16 shows box model outputs from simulations of experiment 1 using four chemical mechanisms (Table S2). These plots can be compared to Fig. S6a, which shows corresponding measurements of DMSO and all oxidized products for experiment 1. The mechanisms capture MSA and sulfate formation to varying degrees, but no scheme captures the observed NO_X-dependence for SO_2 formation. We note that MSA formation from OH addition (panel d) is particularly uncertain; this mechanism uses a computationally calculated rate that exceeds the measured OH + MSIA rate, and assumes all OH-adducts react to form MSA, a reaction that has not been investigated experimentally or computationally.

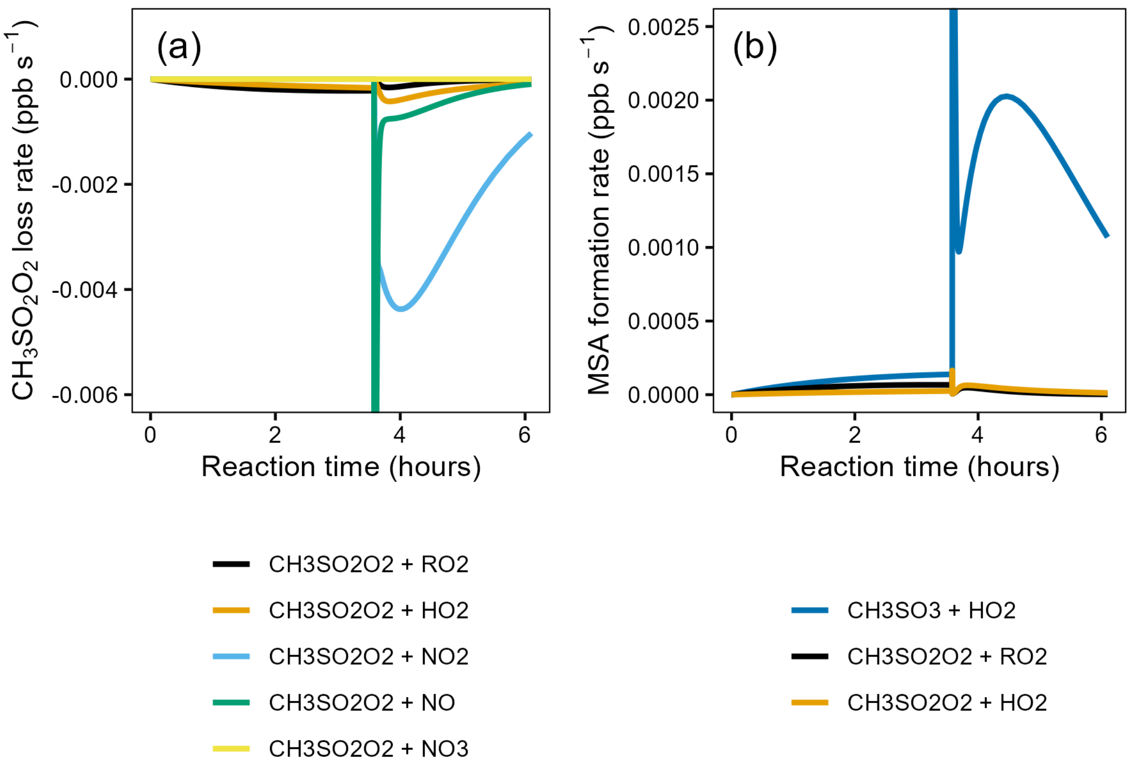

Figure S17: Comparison of rates involved in the production of MSA from a box model representing experiment 1. This model uses default MCM chemistry and the CH_3SO_3 scheme from Table S2. Panel a shows the loss rates of $CH_3S(O)_2O_2$ via bimolecular reactions (note that the equilibrium with CH_3SO_2 is not shown as this reversible reaction far exceeds the rate of these bimolecular reactions). Note that the y-axis is cut off. Panel b shows the production rates of MSA from different pathways for the same experiment.

Figure S17 shows modeled rates associated with the formation of MSA, particularly focusing on the impact of the $CH_3S(O)_2O_2 + RO_2$ reaction. In panel a, which shows the loss rates of $CH_3S(O)_2O_2$, reaction with RO_2 and HO_2 are the main sinks during the first half of the experiment (lower-NO); this suggests that RO_2-RO_2 disproportionation reaction is important under these conditions. During the second half of the experiment (higher-NO), reaction with NO and reaction with NO_2 dominate.

Panel b shows the formation rate of MSA from different reaction pathways. Under lower-NO conditions, the RO_2-RO_2 pathway is the second largest source of MSA, but after HONO is added, the $CH_3SO_3 + HO_2$ pathway dominates. While the RO_2-RO_2 reaction may be important for MSA formation under some conditions, the majority of the MSA observed during these experiments was formed under higher-NO conditions, where the CH_3SO_3 channel is believed to be faster. This conclusion is however subject to uncertainty in the rates and mechanism involved.

S.9.1 Role of $O(^3P)$ in DMSO and DMDS oxidation

While $O(^3P)$ has been shown to be an important oxidant for reduced sulfur compounds in chamber experiments (Van Rooy et al., 2021a, b), its contribution to oxidation under the conditions used in these experiments appears to be negligible, due to lower NO_2 concentrations and lower UV flux ($jNO_2 \approx 0.06$ min^{-1}).

To establish this, expt 1 is modeled using the CH_3SO_3 channel mechanism described in Table S2 and input conditions described in Table 1. After the addition of HONO and NO, when $O(^3P)$ is likely to be highest, modeled $[OH] \approx 2.9 \times 10^6$ molec. cm^{-3}, in good agreement with the OH concentration derived from a fit to the measured DMSO (2.7×10^6 molec. cm^{-3}). Modeled $O(^3P)$ reaches a maximum concentration of 9×10^3 molec. cm^{-3}.

Using the $O(^3P)$ + DMSO rate = 9×10^{-12} cm^3 molec.$^{-1}$ s^{-1} and OH + DMSO rate = 9.4×10^{-11} cm^3 molec.$^{-1}$ s^{-1} at 293 K, DMSO reaction with $O(^3P)$ would make up 0.03% of total reactivity (Burkholder et al., 2020). Assuming similar OH and $O(^3P)$ concentrations for the reaction of DMDS, and literature rates from Burkholder et al. (2020), the DMDS + $O(^3P)$ reaction would make up 0.1% of total reactivity.

S.10 Application of the new AMS quantification method to DMS-derived aerosol

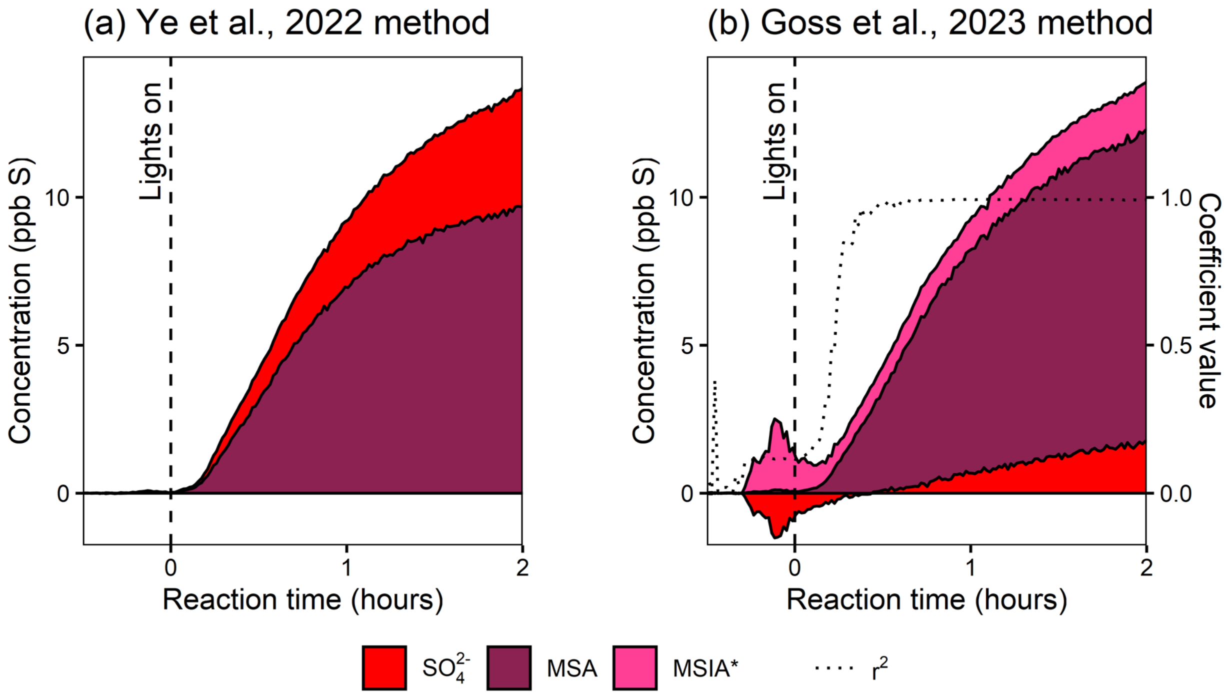

Figure S18: The results of two methods of AMS quantification applied to data from Ye et al., 2022, where DMS was oxidized by OH in the same chamber using HONO as an oxidant precursor. Panel a shows the product distribution using the method from Ye et al., 2022. This approach uses modifications to the frag table, based on Huang et al. (2017). Panel b shows the product distribution using the methods described in this paper (see section S.2). Panel b also shows the r^2 value from the derivation of MSA and MSIA* timeseries using a linear combination. Note that RIE values, collection efficiency, wall loss, and dilution loss corrections have been applied as described in Ye et al., 2022.

As a test case, the AMS quantification method described above is applied to data from Ye et al. (2022). Figure S18 shows the results of the method from Ye et al. (2022) (Panel a), and from this work (Panel b) for an experiment in which dimethyl sulfide was oxidized using HONO as an oxidant precursor. For simplicity, the reference sulfate, MSA, and MSIA spectra used to generate the data shown in Panel b are those taken for this current work, likely adding uncertainty to this quantification.

The calculated aerosol product distribution is similar, but the new method favors MSA as an even more dominant product. In the method used in Ye et al. (2022), the SQUIRREL frag table is modified to apportion signal to MSA based on the signal at m/z 79. The remaining sulfate peaks are assigned to a sulfate timeseries, giving an MSA : sulfate ratio of ~2.4 : 1. When the new method is applied (Panel b), the spectrum from the seed aerosol (present in the chamber before t = 0) is explained by the linear fit to MSIA and MSA with a low but nonzero r^2 value. This results in a positive concentration of MSIA* when none is present. The subtraction of MSIA from the residual spectrum results in a negative sulfate concentration. When the r^2 value

approaches 1 (Panel b), the calculated sulfate concentration is no longer negative. Under this method, the MSA : sulfate ratio increases to ~6.0 : 1 at the end of the experiment.

These results suggest that MSIA* may be a minor but notable additional contributor to rapidly formed DMS-derived aerosol. The partitioning between gas- and particle-phase MSIA/MSIA* observed for this experiment (35 ± 8 % particle-phase (± 1 std. dev.)) agrees closely with that observed for DMSO products. While the presented data show negative sulfate concentrations for the first few minutes of the experiment, it is likely that the use of contemporary reference spectra or a filter based on r^2 could address this. The use of this method could prove useful for future chamber or field measurements where MSA and MSIA are expected to dominate the organosulfur components of the AMS spectrum.

References

Berndt, T., Chen, J., Møller, K. H., Hyttinen, N., Prisle, N. L., Tilgner, A., Hoffmann, E. H., Herrmann, H., and Kjaergaard, H. G.: SO_2 formation and peroxy radical isomerization in the atmospheric reaction of OH radicals with dimethyl disulfide, Chem. Commun., 56, 13634–13637, https://doi.org/10.1039/D0CC05783E, 2020.

Burkholder, J., Sander, S. P., Abbatt, J. P. D., Barker, J. R., Cappa, C., Crounse, J. D., Dibble, T. S., Huie, R. E., Kolb, C. E., Kurylo, M. J., Orkin, V. L., Percival, C. J., Wilmouth, D. M., and Wine, P. H.: Chemical Kinetics and Photochemical Data for Use in Atmospheric Studies, NASA Jet Propulsion Laboratory, 2020.

González-García, N., González-Lafont, À., and Lluch, J. M.: Variational Transition-State Theory Study of the Dimethyl Sulfoxide (DMSO) and OH Reaction, J. Phys. Chem. A, 110, 798–808, https://doi.org/10.1021/jp054424x, 2006.

González-García, N., González-Lafont, À., and Lluch, J. M.: Methanesulfinic Acid Reaction with OH: Mechanism, Rate Constants, and Atmospheric Implications, J. Phys. Chem. A, 111, 7825–7832, https://doi.org/10.1021/jp0722455, 2007.

Hodshire, A. L., Campuzano-Jost, P., Kodros, J. K., Croft, B., Nault, B. A., Schroder, J. C., Jimenez, J. L., and Pierce, J. R.: The potential role of methanesulfonic acid (MSA) in aerosol formation and growth and the associated radiative forcings, Atmos. Chem. Phys., 19, 3137–3160, https://doi.org/10.5194/acp-19-3137-2019, 2019.

Holzinger, R.: PTRwid: A new widget tool for processing PTR-TOF-MS data, Atmos. Meas. Tech., 8, 3903–3922, https://doi.org/10.5194/amt-8-3903-2015, 2015.

Huang, S., Poulain, L., van Pinxteren, D., van Pinxteren, M., Wu, Z., Herrmann, H., and Wiedensohler, A.: Latitudinal and Seasonal Distribution of Particulate MSA over the Atlantic using a Validated Quantification Method with HR-ToF-AMS, Environ. Sci. Technol., 51, 418–426, https://doi.org/10.1021/acs.est.6b03186, 2017.

Koss, A. R., Canagaratna, M. R., Zaytsev, A., Krechmer, J. E., Breitenlechner, M., Nihill, K. J., Lim, C. Y., Rowe, J. C., Roscioli, J. R., Keutsch, F. N., and Kroll, J. H.: Dimensionality-reduction techniques for complex mass spectrometric datasets: application to laboratory atmospheric organic oxidation experiments, Atmos. Chem. Phys., 20, 1021–1041, https://doi.org/10.5194/acp-20-1021-2020, 2020.

Kukui, A., Borissenko, D., Laverdet, G., and Le Bras, G.: Gas-Phase Reactions of OH Radicals with Dimethyl Sulfoxide and Methane Sulfinic Acid Using Turbulent Flow Reactor and Chemical Ionization Mass Spectrometry, J. Phys. Chem. A, 107, 5732–5742, https://doi.org/10.1021/jp0276911, 2003.

Saunders, S. M., Jenkin, M. E., Derwent, R. G., and Pilling, M. J.: Protocol for the development of the Master Chemical Mechanism, MCM v3 (Part A): tropospheric degradation of non-aromatic volatile organic compounds, Atmos. Chem. Phys., 3, 161–180, https://doi.org/10.5194/acp-3-161-2003, 2003.

Van Rooy, P., Purvis-Roberts, K. L., Silva, P. J., Nee, M. J., and Cocker, D.: Characterization of secondary products formed through oxidation of reduced sulfur compounds, Atmospheric Environment, 256, 118148, https://doi.org/10.1016/j.atmosenv.2020.118148, 2021a.

Van Rooy, P., Drover, R., Cress, T., Michael, C., Purvis-Roberts, K. L., Silva, P. J., Nee, M. J., and Cocker, D.: Methanesulfonic acid and sulfuric acid Aerosol Formed through oxidation of reduced sulfur compounds in a humid environment, Atmospheric Environment, 261, 118504, https://doi.org/10.1016/j.atmosenv.2021.118504, 2021b.

Wolfe, G. M., Marvin, M. R., Roberts, S. J., Travis, K. R., and Liao, J.: The Framework for 0-D Atmospheric Modeling (F0AM) v3.1, Geoscientific Model Development, 9, 3309–3319, https://doi.org/10.5194/gmd-9-3309-2016, 2016.

Ye, Q., Goss, M. B., Krechmer, J. E., Majluf, F., Zaytsev, A., Li, Y., Roscioli, J. R., Canagaratna, M., Keutsch, F. N., Heald, C. L., and Kroll, J. H.: Product distribution, kinetics, and aerosol formation from the OH oxidation of dimethyl sulfide under different RO_2 regimes, Atmos. Chem. Phys., 22, 16003–16015, https://doi.org/10.5194/acp-22-16003-2022, 2022.

Zaytsev, A., Breitenlechner, M., Koss, A. R., Lim, C. Y., Rowe, J. C., Kroll, J. H., and Keutsch, F. N.: Using collision-induced dissociation to constrain sensitivity of ammonia chemical ionization mass spectrometry (NH_4^+ CIMS) to oxygenated volatile organic compounds, Atmos. Meas. Tech., 12, 1861–1870, https://doi.org/10.5194/amt-12-1861-2019, 2019.
